# Signaling pathways in vascular function and hypertension: molecular mechanisms and therapeutic interventions

**DOI:** 10.1038/s41392-023-01430-7

**Published:** 2023-04-20

**Authors:** Jun Ma, Yanan Li, Xiangyu Yang, Kai Liu, Xin Zhang, Xianghao Zuo, Runyu Ye, Ziqiong Wang, Rufeng Shi, Qingtao Meng, Xiaoping Chen

**Affiliations:** Department of Cardiology, West China Hospital, Sichuan University, No. 37, Guo Xue District, Chengdu, Sichuan 610041 People’s Republic of China

**Keywords:** Cardiovascular diseases, Molecular biology

## Abstract

Hypertension is a global public health issue and the leading cause of premature death in humans. Despite more than a century of research, hypertension remains difficult to cure due to its complex mechanisms involving multiple interactive factors and our limited understanding of it. Hypertension is a condition that is named after its clinical features. Vascular function is a factor that affects blood pressure directly, and it is a main strategy for clinically controlling BP to regulate constriction/relaxation function of blood vessels. Vascular elasticity, caliber, and reactivity are all characteristic indicators reflecting vascular function. Blood vessels are composed of three distinct layers, out of which the endothelial cells in intima and the smooth muscle cells in media are the main performers of vascular function. The alterations in signaling pathways in these cells are the key molecular mechanisms underlying vascular dysfunction and hypertension development. In this manuscript, we will comprehensively review the signaling pathways involved in vascular function regulation and hypertension progression, including calcium pathway, NO-NOsGC-cGMP pathway, various vascular remodeling pathways and some important upstream pathways such as renin-angiotensin-aldosterone system, oxidative stress-related signaling pathway, immunity/inflammation pathway, etc. Meanwhile, we will also summarize the treatment methods of hypertension that targets vascular function regulation and discuss the possibility of these signaling pathways being applied to clinical work.

## Introduction

Hypertension represents a significant risk factor for cardiovascular and cerebrovascular diseases (CVDs) and remains the primary cause of premature mortality on a global scale.^1^ The estimated number of people aged 30–79-year-old with hypertension doubled from 648 million in 1990 to 1.27 billion in 2019.^2^ The prevention and control of hypertension represent a crucial global public health strategy in the effort to reduce premature mortality from CVDs.^3^

Blood pressure (BP) is defined as the lateral pressure exerted on the walls of blood vessels per unit area during the flow of blood. Hypertension is characterized by an increase in systolic BP and/or diastolic BP. There are two primary factors that directly affect BP: the volume of intravascular fluid and the capacity for vasodilation. The amount of fluid in the blood vessels is mainly related to the everyday intake and output volume. Vasodilatation is the basic function of blood vessels.^4^ The capacity for vasodilation is influenced by vascular elasticity, caliber, and reactivity. Poorer the vasodilatation capacity, higher the BP. The disturbance of vascular contraction and/or relaxation function exerts a great influence on the onset and progression of hypertension. As vasodilatation capacity plays a crucial role in regulating BP, it has garnered significant attention in the field of vascular biology research.^5^

Changes in signaling pathways in vascular endothelial cells (ECs) and vascular smooth muscle cells (VSMCs) are key molecular mechanisms which trigger vascular dysfunction and promote the development of hypertension. Here, we reviewed all key signaling pathways in vascular function and hypertension, as well as their treatment application value in clinical settings.

## Vascular structure and functions of each layer

**Fig. 1:**
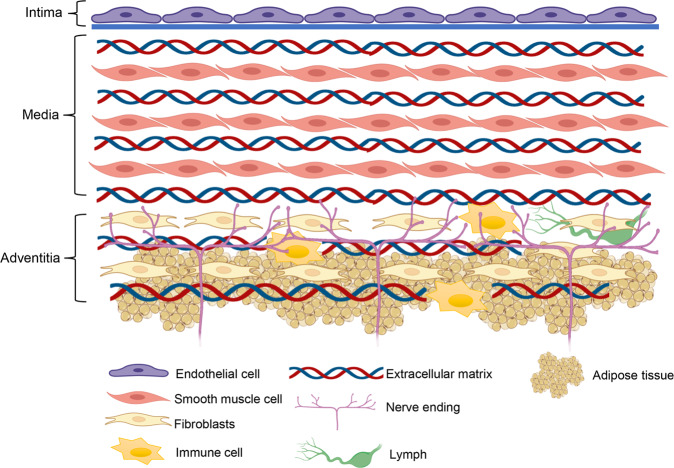
Vascular structure

Blood vessels are composed of three distinct layers, including intima, media, and adventitia (Fig. 1). The intima is mainly composed of ECs, which organize themselves into a continuous monolayer, allowing blood perfusion. On their surface, ECs form a surrounding extracellular matrix (ECM) known as the glycocalyx, which extends beyond the cell surface into the vascular lumen. This structure plays a crucial role in providing a barrier function for ECs, preventing the transmural migration of leukocytes and platelet adhesion.^6,7^ Intima is the “sensor” of vessels, as it can sense various stimuli (such as fluid shear force, cytokines, etc.) in the blood and actively control the degree of vascular relaxation and contraction. This active regulation is mainly achieved through the secretion of various mediators from ECs.^8^ ECs serve not only as a barrier-forming cell population, acting as a responsive interface, but also actively regulate their microenvironment, serving as gatekeepers of organ development, homeostasis, and tissue regeneration.^9^ ECs dysfunction has been regarded as a pivotal mechanism in early pathogenesis of hypertension.^10^ Generalized definition of ECs function or endothelial function involves barrier, secretion, sensation, etc., while in studies with respect to vascular contraction, endothelial function commonly refers specifically to ECs sensing the stimuli from blood and triggering vascular contraction.

Under the ECs is a layer of basement membrane (mainly consisting of type IV collagen and laminins 411^11^) that supports ECs, and under the basement membrane is the middle layer. The medial layer of arteries serves as the load-bearing unit and regulates arterial vascular tone, providing the necessary structural stability for the regulation of blood flow and delivery of oxygen to tissues.^12^ Generally, the farther from the heart, the thinner the middle layer in the arteries. Media layers are absent in the capillaries, which allows adequate exchange of gases and fluids through capillaries.^13,14^ In large (elastic) arteries, elastic fibers interweave with VSMCs, and are surrounded by other ECM.^15^ VSMCs are the main function performer in regulating vasoconstriction and dilation. Once accepting the external signals, VSMCs can transmit the signals by intracellular signaling pathways, thus activating the Actin in VSMCs and triggering cells contraction. Healthy ECM is responsible for maintaining the structure of the middle layer of blood vessels and directly affecting the function and status of VSMCs. Compared with the intima and media, the composition of the adventitia is more complex. The adventitia contains fibroblasts incorporated into a loose collagen extracellular matrix (ECM) enriched in hyaluronic acid. Lymphocytes, nerves, progenitors, adipocytes, and immune cells are also present in the adventitia.^16–18^ Functionally, the adventitia can sense and direct responses to a wide array of stimuli via reciprocal communication among adventitial cells, as well as with cells from neighboring tissues, acting as a biological processing center for the retrieval, integration, and storage.^19^

## Classical signaling pathways in vascular function and hypertension

Abnormal vascular structure is an important cause of hypertension and cardiovascular events.^20–24^ An increase in vascular resistance, largely caused by a reduction in vascular diameter,^25,26^ is a key pathophysiological mechanism contributing to the development of hypertension. The signaling pathways underlying vascular function and hypertension are complex. There are three classical ways to regulate vascular function and BP levels, including calcium signaling pathway, the NO (nitric oxide)-NOsGC (nitric oxide-sensitive guanylate cyclase)-cGMP pathway, and vascular remodeling. Among them, calcium and NO-NOsGC-cGMP signaling pathway are reversible, and vascular remodeling is considered as a pathological change that is difficult to reverse.^27^

### Calcium signaling pathway

**Fig. 2:**
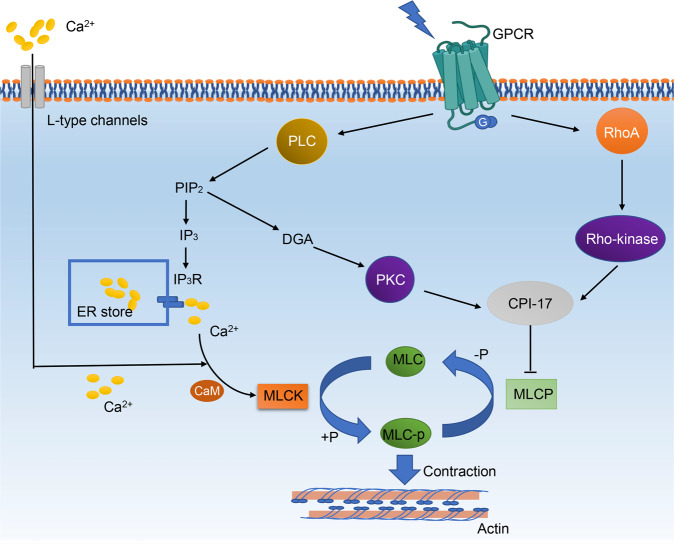
Calcium signaling pathway. CPI-17 molecular mass 17 kDa, DAG diacylglycerol, GPCR G-protein-coupled receptors, IP3 inositol trisphosphate, IP3R inositol trisphosphate receptor, MLC myosin light-chain, MLCK myosin light-chain kinase, MLCP myosin light chain phosphatase, MLC-p phosphorylated myosin light-chain, PLC phospholipase C, PKC protein kinase C

The primary mechanisms regulating the contractile state of VSMCs are changes in cytosolic calcium concentration ([Ca^2+^]c). When vasoconstrictor stimuli are present, intracellular stores and/or the extracellular space mobilize Ca^2+^ to increase [Ca^2+^]c in VSMCs (Fig. 2). The increased [Ca^2+^]c will bind to calmodulin (CaM) and form a complex which can activate myosin light-chain (MLC) kinase (MLCK). Then MLCK will phosphorylate MLC to promote contraction. Conversely, myosin light chain phosphatase (MLCP) can dephosphorylate phosphorylated MLC, triggering vasodilation.^28–31^ One of the important procedures in calcium signaling pathways is the influx of extracellular calcium through voltage-gated Ca^2+^ channels. There exist two main types of Ca^2+^ channels in VSMCs, including the high voltage-activated (HVA) L-type and low voltage-activated (LVA) T-type channels. The primary function of L-type channels is to regulate Ca^2+^ entry for contraction. Nevertheless, it is generally accepted that T-type Ca^2+^ channels (LTCCs) do not play a significant role in arterial vasoconstriction, except possibly in the renal microcirculation.^32^ Many factors, including humoral or neural stimuli, can affect the function (open or close) of LTCCs. And functional regulation of Ca^2+^ channel relies on phosphorylation processes. Except calcium influx, Ca^2+^ release from the internal store (sarcoplasmic reticulum; SR) through the IP3 receptor (IP3R) and the ryanodine receptor (RyR) is also an important way to modulate cellular contraction.^25,29,33^

In addition to the amount of calcium ions in the cytoplasm, contraction is also regulated by calcium-sensitization mechanisms, such as the RhoA-Rho kinase pathway^34,35^ and the PLC (phospholipase C)–DAG (Diacylglycerol)–PKC (protein kinase C) pathway.^36,37^ RhoA is a small GTP-binding protein acting as molecular switcher in signaling pathway, and Rho-kinases (Rho-kinase α/ROKα/ROCK2 and Rho-kinase β/ROKβ/ROCK1) are downstream proteins of RhoA.^38–42^ Rho-kinases contribute to the contraction of VSMCs via Phosphorylation of MLC.^43–45^ Moreover, enhanced RhoA/Rho-kinase (Rho-associated kinase) signaling in VSMCs is considered to be involved in the elevated peripheral vascular resistance observed in clinical hypertension.^36,46,47^ PLC is a phosphodiesterase released through the action of specific phospholipases, and is a downstream product of GPCRs (G-protein-coupled receptors). PIP_2_ (Phosphatidylinositol-4,5-bisphosphate) is a minor phospholipid, which is generated by the hydrolysis of a minor phospholipid in the plasma membrane in response to agonist stimulation. When acted upon by a phosphoinositide-specific PLC (PI-PLC) enzyme, PIP_2_ can generate two intracellular second messengers:^48^ (1) IP_3_ (inositol trisphosphate), which subsequently mobilizes Ca^2+^ from ER stores via IP_3_ receptor;^49^ and (2) DGA, which activates PKC. Activation of PKC can lead to constriction of aorta and BP increasement, via downstream targets of PKC, such as MLCK and CPI-17 (C-kinase potentiated protein phosphatase 1 inhibitor, molecular mass 17 kDa), both of which enhance constriction.^50,51^ Notably, CPI-17 is a smooth-muscle-specific inhibitor of MLCP, which can bind to its catalytic subunit, impending its phosphatase activity and enabling the persistence of contraction. Both PKC and Rho/Rho kinase can induce the enhancement of contractile force via CPI-17.^36,52^

### NO-NOsGC-cGMP pathway

**Fig. 3:**
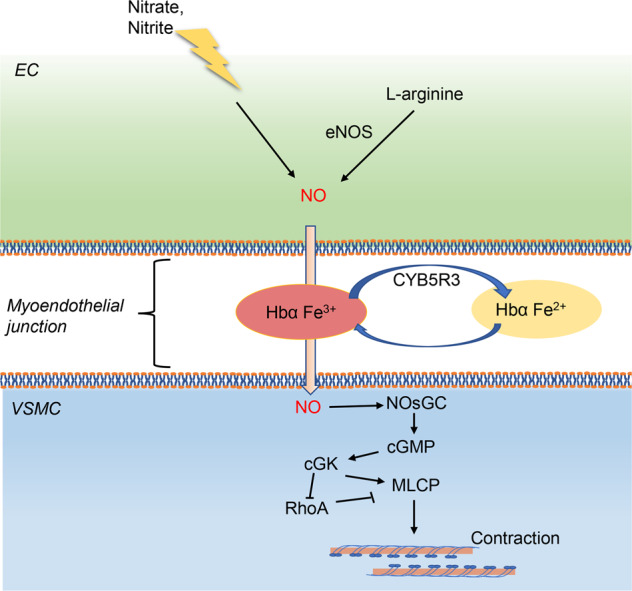
NO-(NOsGC)-cGMP pathway. cGMP cyclic guanosine monophosphate, cGK cGMP-dependent protein kinase, eNOS endothelial nitric oxide synthase, MLCP myosin light chain phosphatase, NO nitric oxide, NOsGC nitric oxide-sensitive guanylate cyclase

The NO-NOsGC-cGMP pathway is closely linked to the contractile function of VSMCs, and its activation precedes the development of hypertension^53–55^ (Fig. 3). The generation of NO in vascular ECs is the beginning of NO-NOsGC-cGMP pathway.^56^ NO production can be stimulated by many chemical factors including L-arginine, nitrate, nitrite, catecholamines,^57,58^ bradykinin,^59^ serotonin,^60^ and physical factors such as fluid shear stress.^61^ And NO can be inactivated by angiotensin II.^62^ There are two pathways by which NO is produced in ECs, including endothelial NOS (eNOS) pathway and eNOS-independent pathway (such as nitrate, nitrite). NO produced from L-arginine by eNOS pathway is the primary source of blood NO.^63,64^ And eNOS knockout has been proven to lead to vascular dysfunction and hypertension.^65^ To be mentioned, a recent study revealed that eNOS did not only contribute to BP regulation in ECs, but also in RBCs. Both the EC and RBC eNOS KOs were significantly hypertensive, and the apply of the NO synthase inhibitor further upregulated BP in these mice.^66^ On the one hand, it indicated the diversity of NO sources in blood vessels. On the other hand, it also suggested that the changes in the quantity of RBCs might affect BP regulation.

NO synthesized in ECs or RBCs can diffuse into VSMCs, and this procedure is regulated by transformation of Fe^2+^ hemoglobin (Hb) α and oxidized Fe^3+^ Hb α. Endothelial Hb α haem iron in the Fe^3+^ state enables NO signaling, while this signaling is terminated when Hb α is converted to the Fe^2+^ state by endothelial CYB5R3 (cytochrome b5 reductase 3).^67^ In VSMCs, NO activates NOsGC, which subsequently generates the second messenger cGMP.^68,69^ cGMP exerts its cellular functions via cGMP-modulated cation channels (cyclic nucleotide-gated [CNG]) and cGMP-dependent protein kinases (cGKs).^70^ One of the cGKs substrate, cGKIα, can bind to and then phosphorylate the myosin-binding subunit of MLC phosphatase, which is crucial for the localization of cGKI near the enzyme it regulates.^71–74^ Besides, transfection of cGKIα could rescue defective Ca^2+^ regulation in cGKI-deficient VSMCs according to studies,^73^ and cGKI activated myosin-bound phosphatase by inhibition of RhoA/Rho-kinase pathway.^75^ All the evidence demonstrated that, cGKIα was also able to relax VSMCs by decreasing the cytosolic Ca^2+^ level and calcium-sensitization, suggesting NOsGC-cGMP pathway had a crosstalk with calcium signaling.

### Vascular remodeling

**Fig. 4:**
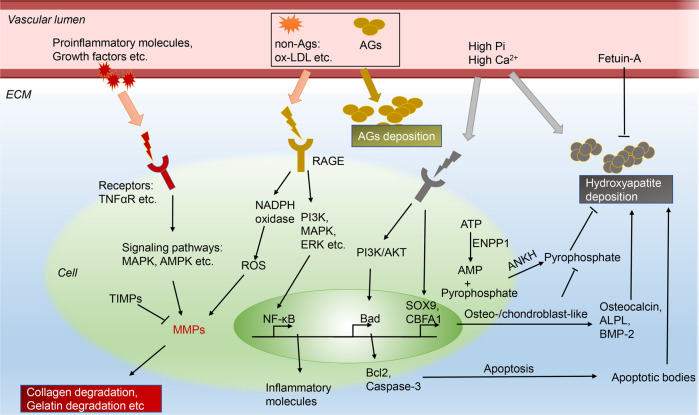
Vascular remodeling pathways. AGEs glycation endproducts deposition, ALPL tissue-nonspecific alkaline phosphatase, AMPK adenosine 5’-monophosphate-activated protein kinase, ANKH transmembrane protein ankylosis protein homolog, BAD bcl2-associated death promoter, BMP-2 bone morphogenetic protein-2, CBFA1 core-binding factor α-1, ECM extracellular matrix, ENPP1 ectonucleotide pyrophosphatase/phosphodiesterase, ERK extracellular-signal-regulated kinase, MMPs matrix metalloproteinases. MAPK mitogen-activated protein kinase, NADPH nicotinamide adenine dinucleotide phosphate, NF-κB nuclear factor κB, HMGB-1 high mobility box group-1, JNK JUN N-terminal kinase, RAGE AGE receptor, ROS reactive oxygen species, ox-LDL oxidized low-density lipoprotein, PI3K phosphatidylinositol-3 kinase, SOX9 chondrogenic transcription factors including SRY-Box 9

Vascular remodeling presents as vascular lumen narrowing, vascular wall thickening, and elasticity loss. It can be put on a par with clinically structural arterial stiffness, which is reflected as pulse wave velocity (PWV) increasement. ECM changes in vessels are pathological conditions which lead to vascular remodeling and hypertension (Fig. 4). No method has been found to reverse the altered ECM to a healthy state currently, and researches in this direction may be the key to breakthroughs in restoring vascular function and curing hypertension. The changes of ECM mainly occur in the media, which may be related to the different SMC phenotypes and different secretory factors from SMCs. The changes of ECM include deposition of excessive collagen and glycation end-products deposition (AGEs), elastic fibers degradation, calcification etc.^26,76–78^

#### MMPs

Matrix metalloproteinases (MMPs) are a group of endopeptidases that depend on zinc and are responsible for breaking down proteins in the ECM. Activated MMPs can deposit collagen, degrade elastin, and then lead to BP increasement.^26,79–83^ At least 28 different types of MMPs are expressed in human tissue. Many types of MMPs are involved in arterial remodeling and BP regulation, including MMP-1,^84–86^ MMP-2,^87–89^ MMP-3,^90,91^ MMP-9^81,89,92,93^ etc. The roles played by MMPs in regulating ECM are not entirely consistent. According to their functions, MMPs can be classified into different types, including collagenases, gelatinases, matrilysins, stromelysins etc.^94–98^ Collagenases, like MMP-1 (interstitial collagenase), can degrade collagen and cleave proMMP-9 into its active form. Gelatinases involve MMP-2 (gelatinase A), MMP-9 (gelatinase B), contributors to degradation of gelatin etc. In addition to the basic functions mentioned above, some MMPs can also function in other ways. For instance, the activation of MMP-2 can lead to calcification^99,100^ and collagen accumulation in the vascular wall.^101^ MMP-9 can cause vascular fibrolysis and enhanced collagen affinity.^95,102^ In addition to acting on vascular remodeling to affect structure, MMPs may also affect BP through other mechanisms. Activation of MMP-2 contributes to an increase in BP by both elevating the levels of big endothelin-1 and reducing the levels of adventitial calcitonin gene-related peptide and endothelial nitric oxide synthase.^103–105^ MMP-1/-9 decreases the density of β (2) adrenergic receptor in arterioles, leading to an increase in arteriolar tone, which also contributes to an elevation in BP.^106,107^

The activity of MMPs is regulated at three levels: proenzyme activation, activity inhibition (tissue inhibitors of MMPs, TIMPs), and gene transcription.^101,108^ During vascular remodeling, the activation of intracellular MMPs is associated with multiple stimulators, commonly including pro-inflammatory signaling molecules (cytokines, interleukins, tumor necrosis factors), growth factors, vasoactive agents (Ang II, ET-1, aldosterone) and their receptors. Signaling pathways involved in regulating MMPs transcription mainly include mitogen-activated protein kinase (MAPK),^101,109,110^ reactive oxygen species (ROS),^111^ adenosine 5’-monophosphate-activated protein kinase (AMPK),^112,113^ extracellular-signal-regulated kinase (ERK),^114^ JUN N-terminal kinase (JNK) etc.^115^, which can either enhance or repress the expression of MMPs. In addition to these general pathways, different types of MMPs may have specific signaling pathways. For example, MMP10 gene transcription is inhibited by HDAC7 (Histone Deacetylase 7) binding to MEF2 (myocyte enhancer factor 2), resulting in endothelial cell–cell adhesion and impaired vascular integrity.^116^

#### AGEs

Reactive byproducts resulting from nonenzymatic glucose–protein condensation reactions, as well as lipids and nucleic acids exposed to reducing sugars, form a diverse group of irreversible adducts known as “Advanced Glycation Endproducts” (AGEs).^117^ AGEs can be formed by glycation of proteins either within cells or in extracellular spaces. This protein glycation consists of a series of complex sequential reactions, known collectively as the Maillard reaction.^118^ Multiple cardiovascular risk factors can result in increase in serum and tissue AGEs, such as diabetes,^119^ hyperlipidemia,^120^ smoking,^121^ etc. According to the sources, AGEs can be classified into endogenous AGEs (intra- and extracellular) and exogenous AGEs taken in from certain foods. The formation of methylglyoxal (MGO) is a major precursor of endogenous AGEs, occurs spontaneously during glycolysis from the triose phosphate isomers glyceraldehyde-3 phosphate and dihydroxyacetone phosphate.^118,122,123^ Other crucial AGEs compounds include glyoxal (GO), 3-deoxyglucosone (3DG), Nε-carboxymethyl-lysine (CML), Nε-carboxyethyl-lysine (CEL), pentosidine, pyrraline, and glucosepane.^118^ Food is the primary sources of exogenous AGEs in human.^124^ Generally, animal fat foods contain more AGEs than plant foods. And cooking at high temperature can increase AGEs in foods.^125^ Moreover, studies have confirmed that controlling dietary habits is an effective way to reduce AGEs in human body.^126–129^

AGEs contribute a lot to vascular remodeling and hypertension. Accumulation of AGEs in ECM leads to the formation of cross-links, which can entrap other local macromolecules.^130,131^ There are three known receptors for AGEs: full-length AGE receptor (RAGE), N-truncated RAGE, and soluble RAGE (sRAGE). sRAGE has two isoforms, including cleaved RAGE (cRAGE) and endogenous secretory RAGE (esRAGE).^132^ Full-length RAGEs and N-truncated RAGEs are multiligand cell bound receptors, while sRAGEs circulate in the blood. AGEs and their receptors are closely associated with vascular function and hypertension. Plasma levels of AGEs are significantly higher in individuals with hypertension compared to those without hypertension and are associated with aortic stiffness independent of age and BP.^133^ Skin AGEs, an important indicator of the current level of accumulated AGEs,^134^ is associated with vascular stiffening independent of age and other cardiometabolic risk factors, not only in individuals with diabetes but also in those in normoglycemic and prediabetic conditions.^135^ Besides, studies reported an inverse correlation between sRAGE and BP,^136,137^ and sRAGE showed its potential in predicting cardiovascular events and/or mortality in diabetics.^138^

Mechanistically, the properties of collagen can be altered through AGEs–RAGE intermolecular covalent bonds or cross-links. Cross-links between AGEs and collagen or elastin increase the extracellular matrix area.^139–141^ On the other hands, cross-linking renders collagen insoluble to hydrolytic enzymes, and collagen linked with AGEs is less susceptible to hydrolytic turnover, making it stiffer.^142,143^ And these factors combined result in vascular remodeling and dysfunction. In intracellular pathway, studies showed interaction of AGEs with full-length RAGE via PI3K, MAPK, ERK1, and ERK2 activates NF-κB (nuclear factor κB), stimulating inflammation and various cytokines secreting.^144,145^ In addition to these cascades leading to inflammation, RAGE activation can increase ROS via NADPH (nicotinamide adenine dinucleotide phosphate) oxidase, and lead to oxidative stress and dysfunction in cells.^146–152^ Notably, RAGE is not only activated by AGEs but also stimulated by other factors including S100 proteins, HMGB-1 (High mobility box group-1), Mac-1 integrin, and ox-LDL (oxidized low-density lipoprotein), thus resulting in vascular dysfunction.^145,151,153–155^ On the contrary, sRAGE can fuction as a decoy receptor for RAGE ligands, effectively binding to them and exerting protective effects against the harmful consequences of the AGE-RAGE interaction on vascular function.^156^

#### Calcification

Vascular calcification refers to the process of calcium deposition in the extracellular matrix of arterial walls. Vascular calcification involves deposition of mineral in the ECM, VSMCs apoptosis and osteogenic transformations, dysregulated expression of mineralization inhibitors, and microvesicle (MV) release et al.^157–159^ There is a mutually reinforcing relationship between vascular calcification and hypertension. Hypertension is a calcification-promoting stressor,^157^ and vascular calcification can result in hypertension through arterial stiffness.^160–162^ Arterial stiffness can initiate media calcification through mechano-sensing pathways, which subsequently exacerbate arterial stiffness.^163^ Though calcification can occur in intima, media, or adventitia,^164,165^ it is commonly considered that medial arterial calcification (MAC) contributes the most to the dysfunction of vascular contraction.^163,166–168^ The pathobiological mechanisms of calcification can be classified into 2 categories: the loss of mineralization inhibitors and the induction of osteogenesis.

MAC is characterized as calcium phosphate depositing in the form of hydroxyapatite in media ECM. Inorganic phosphate is one of the important causes of MAC.^169,170^ Under normal physiological conditions, when the concentrations of calcium and phosphate exceed their solubility limits, the body relies on endogenous calcification inhibitors to prevent the ectopic precipitation of these minerals.^171,172^ Pyrophosphate has been identified as the strongest endogenous inhibitor of mineralization, and it is produced locally by VSMCs.^173–175^ Pyrophosphate can prevent mineralization through several mechanisms, including direct binding to growing crystals, induction of osteopontin expression, and inhibition of Tnap (an issue-nonspecific alkaline phosphatase) activity.^176^ VSMCs are responsible for producing and releasing pyrophosphate into the extracellular space, a process that involves the activation of two proteins: ENPP1 (ectonucleotide pyrophosphatase/phosphodiesterase) and ANKH (the transmembrane protein ankylosis protein homolog).^175,177^ The ENPP1 protein helps break down adenosine triphosphate into AMP (adenosine monophosphate) and pyrophosphate.^178,179^ ANKH protein transports pyrophosphate out of cells to ECM.^180–182^ Besides, VSMCs are also involved in taking up Fetuin-A from the extracellular space, which is a circulating protein that can bind to calcium or hydroxyapatite directly, to inhibit the growth of hydroxyapatite crystals. And VSMCs can produce several other inhibitory proteins, including matrix-Gla protein, osteopontin, and osteoprotegerin. These proteins can be loaded into extracellular vesicles to prevent vascular mineralization.^172,173,183^ Under pathological conditions, including high extracellular phosphate levels and exposure to uremic toxins, the production of calcification inhibitors by VSMCs can be suppressed. This can further result in the release of exosomal vesicles lacking these inhibitors and promoting vascular mineralization. On the contrary, the load of pro-calcific proteins such as ALPL (tissue-nonspecific alkaline phosphatase) is increased.^184,185^ All these combined can lead to the formation of microcalcifications, which can provide a site for the precipitation of calcium phosphate and the subsequent growth of crystals.^185–187^

Apoptosis and phenotypic switching of VSMCs is also critical to calcification. Apoptotic bodies released by VSMCs can potentially serve as nucleating structures for calcium crystal formation.^188,189^ High extracellular phosphate levels can induce apoptosis and necrosis of VSMCs.^183,190^ Activation of pro-apoptotic signaling pathways in VSMCs can occur due to the involvement of a multitude of upstream signaling cascades in response to phosphate. The downregulation of Gas6 (growth arrest-specific gene 6) and its receptor tyrosine kinase Axl, and activation of apoptosis-related BAD (Bcl2-associated death promoter)/Caspase-3 via PI3K/AKT pathway^191–195^ or AMPK (AMP-activated protein kinase),^196–198^ have been thought to be main pathway in apoptosis of VSMCs stimulated by phosphate. Contractile VSMCs converting into osteo-phenotype is also responsible for vascular calcification. Under high extracellular phosphate levels circumstances, VSMCs will undergo a phenotypic switch into osteo-/chondroblast-like cells, and promote vascular mineralization.^199,200^ These phenotypes can express osteogenic transcription factors such as MSX2 (msh homeobox 2), CBFA1 (core-binding factor α-1, also known as RUNX2)^199–201^ or osterix,^202^ as well as chondrogenic transcription factors including SOX9 (SRY-Box 9).^203–205^ Both CBFA1 and SOX9 play critical roles in vascular osteo-/chondrogenic transdifferentiation and calcification.^204,206,207^ The transcription factors, MSX2 and KLF4, have been proven to be the upstream regulators of CBFA1 and SOX9.^208–211^ The expression of osteogenic- and chondrogenic-specific proteins in VSMCs, such as osteocalcin, type I collagen, BMP-2 (bone morphogenetic protein-2), or ALPL, is further induced by osteo-/chondrogenic transcription factors.^170,212^ An increase in ALPL activity is a decisive event in vascular calcification, as ALPL is a key regulator of this process.^186,213^

## Important upstream pathways regulating vascular function and hypertension

### Renin-angiotensin-aldosterone system

**Fig. 5:**
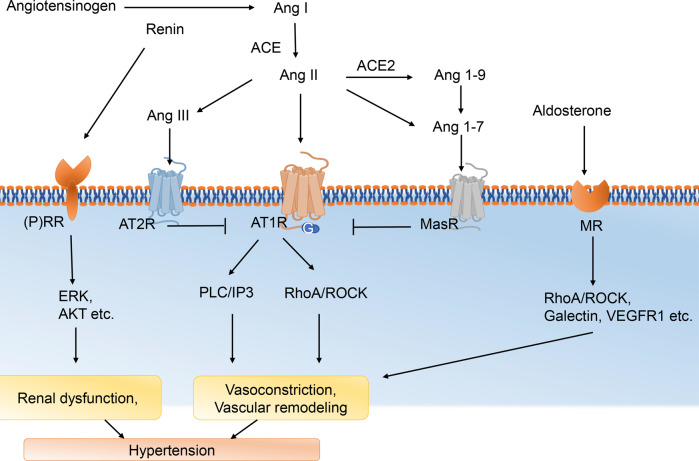
Renin-angiotensin-aldosterone system. ACE angiotensin-converting enzyme I, ACE2 angiotensin-converting enzyme 2, Ang I angiotensin I, AT1R Ang II type 1 receptor, Ang (1–7) angiotensin (1–7), MR mineralocorticoid receptor, (P)RR (pro)renin receptor, VEGF1R vascular endothelial growth factor type 1 receptor

Renin-angiotensin-aldosterone system (RAAS) plays a critical role in the regulation of BP. In the RAAS, angiotensinogen and downstream peptides are the main stimulators regulating vasoconstriction (Fig. 5). Angiotensinogen mainly produced by the liver is cleaved by renin into angiotensin I (Ang I). Ang I is cleaved by angiotensin-converting enzyme I (ACE1) to produce angiotensin II (Ang II) or cleaved by angiotensin-converting enzyme 2 (ACE2) to produce Ang (1–9). Ang II exerts various physiological and pathophysiological effects, including vasoconstriction and sodium/water retention, via activation of Ang II type 1 receptor (AT1R) signaling.^214^ Moreover, Ang II or Ang (1–9) can be converted to angiotensin 1–7 [Ang (1–7)],^215^ and Ang (1–7) can cause vasodilation and lower BP by binding to Mas receptor (MasR).^215–218^ Those peptides can have actions on endothelial cells, SMCs, and adventitia cells in vessels. Various peptides activating the receptors on different cells can result in different effects, including apoptosis and endothelial barrier damage for endothelial cells, contraction of SMCs, and fibrosis of atria.^219–221^ In addition, recent study reported the role of Ang (1–12) in the process of hypertension, the angiotensin family is still under development.^222^

#### Angiotensin-related receptors

The signaling pathways of RAAS in cells begin with the activation of various receptors, and the diverse effects of RAAS can be attributed to the activation of different receptors.^223,224^ Angiotensin-related receptors are the main executors of the RAAS system in regulating vasoconstriction and BP. AT1R is a member of the GPCR (G protein-coupled receptor) family and is expressed in various cells including VSMCs, endothelium, cardiomyocytes, etc. Ang II/AT1R play a central role in regulating BP. In VSMCs, AT1R can be stimulated by Ang II and interact with a heterotrimeric G protein including G_q/11_ and G_12/13_. G_q/11_ can subsequently activate PLC-IP3-Ca^2+^-sensitive-MLCK signaling. While G_12/13_ will active PKC-RhoA/Rho kinase-mediated inhibition of MLCP.^214^ In addition, study showed that Ang II/AT1R inhibited the MLCK transcription by blocking the Notch signaling in VSMCs.^225^ On the contrary, Ang (1–7)/MasR is a bioactive peptide that can exert diverse effects, many of which are contrary to those induced by Ang II/AT1R.^226–228^ The Ang 1–7/MasR axis activation acts as a counter-regulator for the effects mediated by Ang II/AT1R, but the exact mechanism remains unclear. A recent study has found that Ang 1–7/MasR axis exerts vasodilation through two mechanisms: a telomerase-dependent manner and direct increase of telomerase activity in human endothelium.^229^ Since increased telomerase activity can elevate NO production and endothelial nitric oxide synthase expression,^230^ it is supposed that Ang 1–7/MasR-mediated vasodilation may via NO-(NOsGC)-cGMP pathway.

In addition to Ang II/AT1R and Ang 1–7/MasR, other peptides or receptors are also involved in BP regulation. Upregulation of vascular and plasma ACE2, along with increased plasma Ang 1–9 levels, can exibit a potent antihypertensive effect via RhoA/Rho kinase inhibition, without an increase in Ang 1–7 levels.^231^ The AT2R can promote the production of NO and cGMP by two different mechanisms, dependent or independent of the production of bradykinin (BK) via BK B2 receptors.^232–234^ ANG IV, cleaved from Ang (1–9), can bind to AT4R and cause vasorelaxation via eNOS, too.^235^

#### Angiotensin-unrelated receptors

The (pro)renin receptor ((P)RR) is thought to enhance the activity of the tissue renin–angiotensin system by binding to renin or prorenin, and it can activate intracellular tyrosine-phosphorylation-dependent pathways independently of RAAS.^236^ However, whether (pro)renin/(P)RR contributes to hypertension in human is controversial. A study showed that in VSMCs, prorenin can induce ERK phosphorylation via (P)RR-mediated activation of tyrosine kinase. This can subsequently lead to a vascular remodeling via MEK,^237^ independently of the production of angiotensin II or the activation of its receptors. Another study demonstrated that (P)RR was essential for VSMCs survival and downregulation of vascular inflammation, through maintaining normal function of the vacuolar H(+)-ATPase in Wnt signaling.^238^ And the deletion of (P)RR did not affect ambulatory BP levels in murine.^238^ To elicit intracellular signaling in vitro, much higher concentrations of (pro)renin are required than those observed in physiological plasma levels. The effects observed in animals overexpressing prorenin could be solely due to the generation of angiotensin, possibly without requiring a receptor.^239^ Compared with hypertension, it is now considered that (P)RR and the downstream pathway play more important roles in cell survival. (P)RR knockout, even tissue-specific, is lethal compared to other RAS components knockout, suggesting that (P)RR has an important function independent of (pro)renin.^240,241^ To be mentioned, in clinical studies, the addition of direct renin inhibitors (such as aliskiren) to ARB therapy did not lead to improved renal or cardiovascular outcomes. Conversely, it was associated with a higher incidence of adverse effects compared to ARB therapy alone.^242^ According to that, the prospect of targeting (P)RR in the treatment of hypertension is not as good as AT1R. Another angiotensin-unrelated receptor is mineralocorticoid receptor (MR). The mechanism by which MR affects BP involves its binding with aldosterone and subsequent retention of sodium. Mice with MR deficiency in VSMCs exibit decreased vascular myogenic tone, reduced contraction in response to agonists, as well as decreased expression and activity of L-type calcium channels.^243^ MR mediates vascular remodeling via several signalings, including RhoA/ROCK, placental growth factor (PLGF), vascular endothelial growth factor type 1 receptor (VEGF1R), and galectin signaling et al.^244^

### Redox signaling pathway

**Fig. 6:**
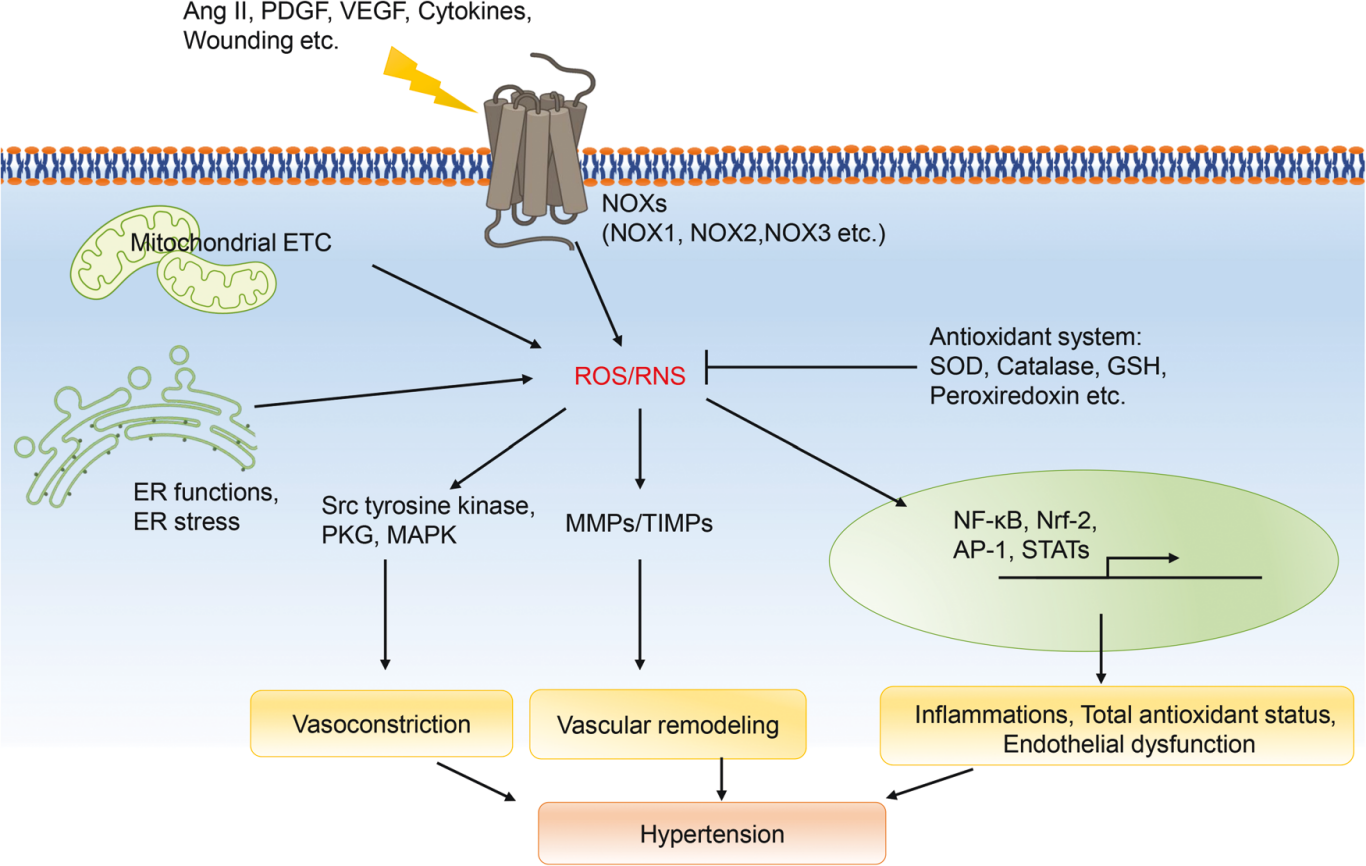
Redox signaling pathway. AP-1 activator protein 1, ECT electron transport chain, ER endoplasmic reticulum, NOXs NADPH oxidases, ROS/RNS reactive oxygen/nitrogen species, SOD superoxide dismutase, STATs signal transducers and activators of transcription, PDGF platelet-derived growth factor, PKG protein kinase G, VEGF vascular endothelial growth factor

Cellular reduction/oxidation (redox) signaling pathway is responsible for regulating a wide range of cellular functions, including but not limited to homeostasis, differentiation, proliferation, and apoptosis.^245^ Cellular oxidative stress occurs when reactive oxygen/nitrogen species (ROS/RNS) is produced. Generally, ROS/RNS substances include hydrogen peroxide (H_2_O_2_), hydroxyl radicals (HO), superoxide anion radicals (O_2_^–^), nitric oxide (NO), nitrogen dioxide (NO^-^), peroxynitrite (OONO^−^), dinitrogen trioxide (N_2_O_3_), and nitrous acid (HNO_2_) radicals.^246^ Redox signaling is primarily characterized by an oxidation–reduction reaction or covalent adduct formation that occurs between the sensor signaling protein and second messenger.^247^ Multiple transcription factors and enzymes are all redox-sensitive.^248^ Redox signaling has been firmly established in vascular function and hypertension (Fig. 6). Activation of redox signaling increases vascular tone by influencing the regulatory role of ECs, and by directly affecting the contractility of VSMCs.^249–252^ Oxidative modifications affect multiple kinases, such as Src tyrosine kinase,^253^ ASK-1,^254^ PKG (protein kinase G),^255^ and MAPK pathway, by which regulating vascular contraction.^256,257^ As mentioned above, ROS is also an important factor in the activation of MMPs, leading to vascular remodeling and BP increase. Furthermore, the expression and function of various transcription factors, including NF-κB, Nrf-2, AP-1 (activator protein 1), STATs (signal transducers and activators of transcription) etc., are affected by ROS. The activation of these factors can lead to inflammation, total antioxidant status, endothelial dysfunction, and hypertension.^249,258–261^

Endogenous ROS/RNS are mainly produced through mitochondrial electron transport chain (ECT),^262–264^ enzymes NADPH oxidase,^265^ and other sources including xanthine oxidase,^266^ peroxisomes,^267^ endoplasmic reticulum^268^ etc. At the same time, there is a scavenging system in redox signaling to maintain ROS/RNS balance in the body.

#### Mitochondrial electron transport chain

In physiological conditions, superoxide is generated as a side product of electron transportation during oxidative phosphorylation in SMC mitochondria.^269,270^ Although most superoxides stay in mitochondrial matrix, some can escape to the intermembranous space and cytosol, via anion channels when the superoxides generation is excessive.^271^ This is one of the main sources of superoxides contributing to redox signaling activation.

#### NADPH oxidases

NADPH oxidases (NOXs) are enzymes that produce superoxides by transporting electrons from NADPH to molecular oxygen. NOXs are well-known as important sources of ROS in blood vessels.^272^ NOXs system is composed of NADPH oxidase constituents (such as Nox1, Nox2, Nox4, and Nox5) and cytosolic proteins. Activation of each constituent involves distinct regulatory mechanisms and signaling pathways, which can be attributed, in part, to the variety of cytosolic regulatory subunits, including p47phox, p67phox, Rac, Noxo1, and Noxa1.^251,273,274^ Hypertensive animal models, such as Ang II-induced hypertensive rats,^275^ SHR,^276^ DOCA-salt hypertensive rats, and two-kidney two-clip renovascular hypertensive rats, have been shown to exhibit increased expression and activity of NADPH oxidase or its cytosolic subunits.^277–279^ The activation of vascular NADPH oxidases through PKC, Src^280^, or CyPA^281^ dependent pathways is an important mechanism by which Ang II functions in the body. Besides, NOX1-derived reactive oxygen species regulate cell-surface AT1R expression by mechanisms like caveolin phosphorylation,^282^ which suggests that there is positive feedback between Ang II and NOXs. Other stimulants include PDGF, VEGF, cytokines, wounding^254,283–285^ etc.

#### Endoplasmic reticulum

There is growing recognition of the important role that the endoplasmic reticulum (ER) palys in redox pathophysiology, summarized in another review^249^ as: (i) ER enzymes, like ER oxidoreductin (Ero1) and its thiol redox partner protein disulfide isomerase (PDI), have a role in the generation of ROS, (ii) ER is responsible for the synthesis, maturation and post-translational modification (glycosylation, phosphorylation, oxidation) of p22phox and Nox, (iii) Nox activity is promoted by the interaction between Nox and the ER chaperone PDI, (iv) some Noxes, especially Nox4, are active in ER, (v) The communication between ER and mitochondria occurs through mitochondria-associated ER membranes, which facilitates the exchange of Ca^2+^ and ROS between the compartments, and (vi) ER plays a critical role in redox protein folding and stress responses.

ER stress results from a disruption in the ER protein-folding capacity, leading to the accumulation of unfolded and misfolded proteins.^286^ There is a suggestion that ER stress plays a significant role in the synergistic effects of hypertension and target organ damage.^287–289^ Expressions of key molecules increase in ER stress signaling pathway, such as ATF6 (activating transcription factor-6), IRE1 (inositol requiring enzyme 1), PERK (PKR-like eukaryotic initiating factor α kinase), XBP1s (X-box-binding protein 1), ATF4 (activating transcription factor-4), and CHOP (C/EBP homologous protein) etc., leading to activation of redox signaling and hypertension.^289^

#### Antioxidant system

In addition to increased ROS/RNS production, decreased scavenging ability also contributes to oxidative stress. The vascular system have several antioxidant systems, such as the superoxide dismutase (SOD) family, catalase, the glutathione (GSH) system, thioredoxin, peroxiredoxin, selenoproteins, and ROS scavengers such as vitamins A, C, and E.^290–293^ In hypertensive patients, antioxidant substances, including SOD, catalase, and GSH peroxidase etc., are significantly lower in the whole blood or peripheral mononuclear cells, when compared to normotensive individuals. After antihypertensive treatment, all these parameters can be restored.^294–296^ In animal studies, it has been observed that inhibiting GSH synthesis leads to an elevation of BP in normotensive rats,^297^ and knockout of extracellular-SOD (EC-SOD) in mice results in elevated baseline BP without any other treatment.^298^ In addition, partial deficiency of SOD2, which is the mitochondrial SOD isoform, induced spontaneous hypertension in aged mice and accelerated the development of high salt-induced hypertension.^299^ These results suggest that reduced antioxidant capacity alone is enough to cause vascular dysfunction and hypertension. Catalase is another important antioxidant enzyme. Catalase facilitates a two-step reaction in which it breaks down two molecules of hydrogen peroxide into one molecule of oxygen and two molecules of water. Accumulation of catalases attenuates the progression of vascular remodeling and hypertension by reducing oxidative stress.^300–303^

### Immunity/inflammation pathway

**Fig. 7:**
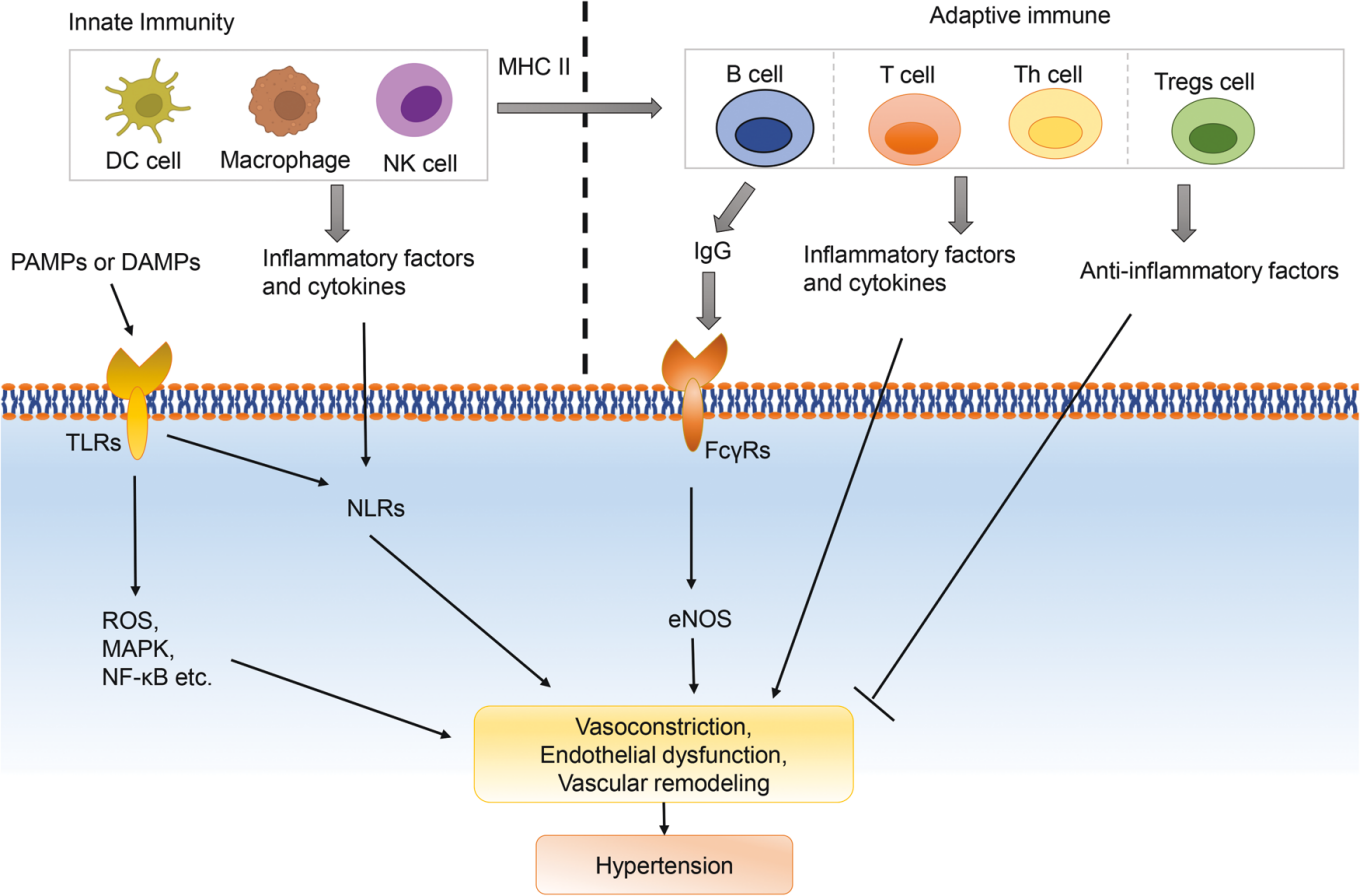
Immunity/Inflammation pathway. DAMPs Damage-Associated Molecular Patterns, FcγRs Fcγ receptors, NLRs NOD-like receptors, PAMPs Pathogen-Associated Molecular Patterns, TLRs Toll-like receptors

The majority of components involved in the Immunity/inflammatory responses circulate through the blood and vasculature. And low‐grade immune response plays an important role in the initiation and maintenance of elevated BP.^304–306^ Immune responses can occur in the intima, media, and adventitia of blood vessels. The luminal and microvascular endothelial cells in the intima and adventitia play a critical role in the recruitment and activation of leukocytes, which are commonly involved in the pathophysiological processes of hypertension and target organ damage.^307^ The vascular media, in contrast, is often unaffected by immune-mediated disorders.^307^ In arteries, the inflammatory response can be initiated by humoral, vasoactive hormones, mechanical factors, metabolic factors, epigenetic dysregulation, autonomic nervous system etc.^308–311^ Current studies have shown that both innate and adaptive immunity are involved in the pathogenesis of hypertension^306,312–314^ (Fig. 7).

#### Innate immunity

The innate immunity serves as the initial defense against infectious agents and also contributes significantly to the development of sterile inflammation.^315^ APCs (activation of antigen presenting cells) or PRRs (pattern recognition receptors) is the initiating step of innate immunity. MHC II (major histocompatibility complex II) in APCs can initiate activation of T or B lymphocytes and lead to adaptive immunity.^312^ While PRRs, activated by PAMPs (Pathogen-Associated Molecular Patterns) and DAMPs (Damage-Associated Molecular Patterns), mainly including TLRs (Toll-like receptors) and NLRs (NOD-like receptors), directly activate related pathways to trigger endothelial dysfunction or vasoconstriction. TLRs are a well-characterized family of membrane-bound PRRs which are expressed on the cell membrane in macrophages, dendritic cells, and mast cells.^316–319^ Studies demonstrated TLR4 expression was upregulated in experimental models of Ang II and L-NAME-induced atrial hypertension.^320–322^ TLR4 inhibition contributed to alleviating vascular contractility, vascular inflammation, and oxidative stress in SHRs, preventing the development of experimental hypertension.^320,323,324^ Stimulation of TLR4 leads to the activation of signaling pathways or transcription factor in VSMCs such as ROS, MARK, NF-κB etc.^325–327^ TLR9, which recognizes circulating mitochondrial DNA, is also upregulated in the circulation of SHRs. Inhibition or knockout of TLR9 in mice can result in a reduction in systolic BP, which may be via the cardiac autonomic and baroreflex regulation.^328,329^ Another family of PRRs are intracellular NLRs, with the NLRP3 inflammasome being the most characterized. Activation of NLRP3 inflammasome (including NLRP3 sensory component, the adaptor protein ASC) is a powerful mediator of inflammatory response via the effector protein caspase-1, and plays a pivotal role in vascular diseases.^330–332^ Study showed that NLRP3 inflammasome activation contributed to VSMC phenotypic transformation and proliferation in hypertension.^333^ And CaSR (calcium-sensing receptor)-mediated activation of the NLRP3 inflammasome in VSMCs is an important regulator of aortic remodeling in SHRs induced by Ang II.^334^

#### Adaptive immunity

T and B lymphocytes are the characterized cell types of the adaptive immune system. Animal studies proved that, inhibition of the maturation process of T and B cells by knockout of the Rag1 (recombination activating gene1) in vivo alleviated Ang II-induced and salt-sensitive hypertension.^335,336^ Besides, genetic knockout of the CD247 or receptor Axl (tyrosine kinase TAM family member) gene in mice attenuated salt-sensitive hypertension by attenuating glomerular and renal tubular damage or improving endothelium-dependent vasorelaxation.^337–339^ Notably, study demonstrated that, it was CD8^-/-^ mice but not MHCII^-/-^ or CD4^-/-^ mice that showed a blunted response in Ang II and DOCA/salt-Induced hypertension, which suggested that the role of specific T cell subtypes might be different in hypertension development.^340^ Similarly, knocking out B cell activating factor receptor (BAFF-R) also attenuated Ang II-induced BP elevation, and adoptive transfer of B cells into BAFF-R^-/-^ mice restored Ang II-induced hypertension.^341^ The mechanisms by which T and B cells trigger hypertension have not been fully elucidated. A study has suggested that hypertension-induced sodium excretion via eNOS- and COX-2 (cyclooxygenase-2)-dependent pathways in kidneys is facilitated by the absence of lymphocyte activity, which may in turn protect against hypertension.^342^ In vascular ECs, IgG released from B cell binding to Fcγ receptors (FcγRs) and inhibiting the activity of eNOS synthase, which contributing to obesity-induced hypertension.^343^

In addition to T and B cells, other subtypes of T cells, mainly including T helper (Th) cells and regulatory T cells (Tregs), also contribute to the progression of hypertension by releasing pro-inflammatory factors. Th 1 cells produce IFNγ, IL-2, and TNFα; Th 2 cells produce IL-4 (interleukin-4), IL-5, IL-9, and IL-13; Th 17 cells secrete IL-17, IL-21, and IL-22. These inflammatory factors are all thought to be associated with hypertension.^27,344–350^ Tregs are a small subset of immune cells that play a crucial role in curbing excessive immune activation and maintaining immune homeostasis.^351–353^ A decreased number and impaired function of Tregs cells have been observed in various cardiovascular diseases, including hypertension.^354^ In contrast to T cells or Th cells, differentiation and proliferation of Tregs can increase local production of anti-inflammatory cytokines (such as IL-10 and TGF-β) and reduce plasma inflammatory cytokines (such as IFN-γ, IL-6, and TNF-α) levels, which thereby attenuates the vascular immune-inflammation, vascular oxidative stress, and endothelial dysfunction, preventing against hypertension.^355–359^ Interestingly, studies have shown that a decrease in Tregs significantly increases BP only in females, thereby eliminating the sex difference in the BP response to DOCA-salt,^360^ which supports the notion that the immune system contributes to sex differences in hypertension. It is worth noting that while hypertension and inflammation are physiologically connected, the impact of therapies that specifically target inflammation on BP still requires further evidence from clinic trials. IL-1β is an upstream inflammatory factor of IL-6 and CRP (C-reactive protein). IL-1β, IL-6, and CRP are considered to be closely involved in the progression of hypertension.^27,361–363^ However, a large clinical study have shown that the use of IL-1β antibody did not reduce the incidence of hypertension.^364^ Thus, the use of anti-inflammatory therapy to lower BP requires more in-depth study.

### Sympathetic dysregulation

**Fig. 8:**
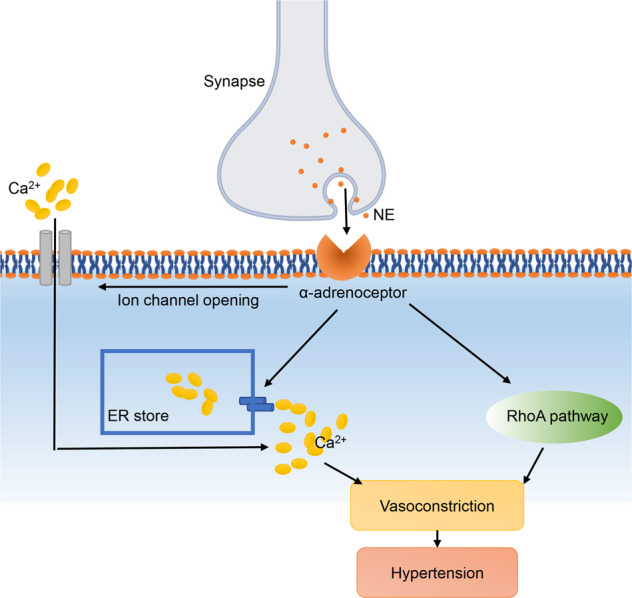
Sympathetic pathway. ER endoplasmic reticulum, NE norepinephrine

The regulation of bodily functions by the sympathetic system relies on the establishment and precise connections between postganglionic sympathetic neurons and peripheral organs distributed throughout the body.^365,366^ Sympathetic nervous system dysfunction contributes a lot to the development of hypertension^367–372^ (Fig. 8). The activation of muscle sympathetic nerve activity (SNA) is the key mechanism of sympathetic dysregulation leading to hypertension. Many forms of high BP are associated with an increase in muscle SNA, including essential hypertension,^373–375^ renovascular hypertension^376,377^ and pregnancy-induced hypertension^373,378^ et al.

Delivering electrical stimulation in bursts to sympathetic nerves in blood vessels can cause significant vasoconstriction. It is closely associated with norepinephrine (NE)-induced contraction of vascular smooth muscle, with NE being released by postganglionic neurons.^379,380^ Adrenergic receptors are distributed on the membranes of most effector cells innervated by the postganglionic fibers. Vascular SMCs have two types of adrenergic receptors, α and β. NE binding to α adrenergic receptors can cause VSMCs contraction; and it binding to β adrenergic receptors (mostly β2 receptors) can cause VSMCs relaxation. NE prefers to bind to α adrenergic receptors rather than β receptors, so it causes a vasoconstrictive effect when the vasoconstrictor fibers are excited.^381^ α adrenergic receptors on the vessels are divided into α1 and α2 receptors. And α-adrenoceptors can be classified into different subtypes, too.^381–383^ Both α1 and α2-adrenoceptors are crucial in the control of vascular tone. There also exists a synergistic interaction between α1 and α2-adrenergic receptors.^384^ Following increased muscle SNA, NE binds both α1 and α2-adrenergic receptors on vascular smooth muscle, which can lead to vasoconstriction by increased [Ca^2+^]c and MLC phosphorylation, as well as activation of the Rho-associated kinase calcium sensitization pathway.^385–387^

## Other signaling pathways afftecting vascular function and hypertension

### Signaling pathways in cell identity

With the widespread utilization of single-cell sequencing (scRNA-seq) technology, our understanding of the intrinsic properties of cells has shifted from single-dimensional descriptions to multi-dimensional and high-resolution depictions. Describing cell identity by key concepts such as phenotype, lineage, and state, we gain a deeper understanding of the mechanisms by which the cells can play different roles in different environments. In the vascular wall, phenotypic conversion, lineage origin, and cellular state of ECs and SMCs may all affect the vascular functions directly.

There are two states of ECs, activated or quiescent. Activated ECs secrete MMPs, and undergo proliferation and migration, which are often closely associated with angiogenesis.^8,388,389^ While quiescent ECs organize themselves into a continuous monolayer and allow perfusion with blood.^8,390^ The production of S-2-hydroxyglutarate (S-2HG), stimulated by activated FOXO1 (transcription factor forkhead box O1), plays a key role in promoting a quiescent endothelial state.^391,392^ The different states of ECs can determine the longitudinal extension of blood vessels. However, no studies have reported the role of activated or quiescent ECs in vasocontriction or BP regulation yet. In addition, there exists some condition where ECs can transform into mesenchymal cells, which is called Endothelial to Mesenchymal Transition (EndMT). A variety of cellular properties will change in ECs after EndMT, such as increased permeability, enhanced migration, upregulated expression of some mesenchymal cellular markers, and enhanced ability to secret enzymes and to change the ECM environment.^393,394^ TGF-β (Transforming Growth Factor-β) signaling pathtway is now considered as the most significant pathway in EndMT regulation. After binding to the receptors, TGF-β can participate in the EndMT via Smad (Small Mother against Decapentalegic) and non-Smad (MAPK, ERK, etc.) pathways.^394,395^ Other signaling pathways, such as NOTCH and Wnt (Wingless-Related Intergration Site) pathway, can also induce EndMT.^394,395^ Just a few studies reported the association of EndMT with BP alterations. It was indicated that ox-LDL could promote the production of TGF-β via Lox-1/PKC-α/MMP9, and it also could induce EndMT via SMAD2/SMAD3, resulting in increased BP.^396^ And this is possibly one of the crucial mechanisms by which high blood lipids induce BP increase. Some stimulators closely associated with hypertension, such as Angiotensin II^397^ and AGEs,^398^ were also confirmed to induce EndMT, thus leading to BP increase. In addition, pro-inflammatory factors, such as tumor necrosis factor-α (TNF-α) and interleukin-1β (IL-1β), have been reported to induce EndMT in human primary aortic ECs and to promote vascular calcification by downregulating BMPR2.^399^ A recent study used scRNA-seq to detect the heterogeneity and state of arterial cells in mice with salt-induced hypertension, and found that, EndMT was more commonly observed in arterial cells in hypertensive mice, compared with the control group. And this suggested that the occurrence of hypertension might be directly related to EndMT.^400^

VSMCs are classically characterized by the expression of four contractile- or contraction-associated proteins: TAGLN (smooth muscle 22 α/transgelin), ACTA2 (smooth muscle α-actin), MYH11 (smooth muscle myosin heavy chain 11), and CNN1 (H1-calponin).^401^ With the help of scRNA-seq, six phenotypes of VSMCs have been identified, including contractile phenotype, mesenchymal-like, fibroblast-like, macrophage-like, osteo-/chondroblast-like, and adipocyte-like phenotypes, each directly affecting the ECM with their own unique characteristics.^402–405^ KLF4 (Krüppel-Like Factor 4) is considered as a key transcription factor in phenotype regulation.^405^ Although, it is theoretically possible for VSMCs switching from a contractile phenotype to other phenotypes, which may affect the contractile function of the arteries, the role of different phenotypes in arterial function and hypertension is still unknown. At present, it is clear that the osteo-/chondroblast-like phenotype is involved in the formation of arterial calfication. In a study of salt-induced hypertensive mice,^400^ VSMCs were divided into 3 groups (SMC 1, SMC 2, and SMC 3) according to their markers. SMC 1 was observed to be the most expressed group in both the hypertensive and control group. The contractile phenotype-related gene expression in SMC 1 was lower in hypertensive group than that in control group. However, The expression of KLF4 was significantly higher in hypertensive group. This indicated that part of the SMCs in aorta experienced phenotypic conversion in the salt-induced hypertensive mice, which might be associated with the occurrence of hypertension. Unfortunately, the study did not further explore which phenotype is the dominant phenotype in hypertensive arteries. Nevertheless, according to our own research data (unpublished), long-term high blood lipids level can lead to arterial remodeling and increased arterial stiffness. And the pathological mechanism may be that, induced by high blood lipids, VSMCs can convert from contratile phenotype into macrophage-like phenotype, resulting in changes in ECM composition and decreased cell contractility. However, the roles of other phenotypes, such as fibroblast-like and adipocyte-like phenotype, in vascular function and BP regulation remain to be further explored.

### Vascular fibrosis and TGF-β/Smad signaling pathway

Fibrosis refers to the excessive accumulation of connective tissue components in an organ or tissue. Vascular firobrosis is caused by the excessive deposition of the ECM components (especially fibronectin and collagen), which can result in a decrease in lumen diameter and thickening of arterial wall,^406,407^ and further lead to arterial stiffness and hypertension.^101,408^ Although whether the two concepts of vascular fibrosis and vascular remodeling should be unified is inconclusive for the time being, they are almost consistent from the perspective of pathological changes and diseases caused. TGF-β and downstream Smad is the key signaling pathway that promotes tissue fibrosis.^407,409,410^ In various cells in blood vessels, such ECs, VSMCs, and fibroblasts in adventitia, the activation of TGF-β/Smad pathway can stimulate the synthesis of fibronectin and collagens, and promote their deposition in ECM, thus resulting in vascular fibrosis.^407,411,412^ CTGF (connective tissue growth factor) is a chaperon protein that has a synergistic effect with TGF-β to promote fibrosis.^413^ Studies showed that the activation of TGF-β signaling pathway would further upregulate CTGF gene expression,^414^ which indicated that CTGF played an important role in regulating TGF-β pathway in a positive feedback way. PAI-1 (plasminogen activator inhibitor-1) is an inhibitor of serine protease, urinary plasminogen activator (uPA) and tissue plasminogen activator (tPA). The activation of TGF-β/Smad pathway can upregulate PAI-1 gene expression. And the increase of PAI-1 in ECM will inhibit tissue proteolytic activity and collagen degradation, thus leading to protein accumulation in ECM, vascular fibrosis, and hypertension.^415,416^ Moreover, as mentioned above, TGF-β/Smad pathway is also a crucial pathway which promotes EndMT. It is also worth noting that, AGEs can activate Smad via MAPK pathway, independently of TGF-β,^417^ which indicates that AGEs may also be one of the causes of vascular fibrosis.

### Apelin/APJ signaling pathway

A new class of transmembrane receptor, APJ (putative receptor protein related to the angiotensin receptor), was first discovered in 1993 by O’Dowd. It has homology as high as 30% with AT1R, while it does not bind to angiotensin II.^418^ In 1998, an endogenous ligand for APJ, called apelin, was extracted from cow stomach by Tatemoto et al.^419^ Apelin is type of polypeptide hormone. APJ exists in a variety of cells such as ECs and VSMCs, and is widely expressed in large blood vessels and the vascular system of various organs.^420–423^ Apelin/APJ mediates vasodilation primarily by activating the eNOS/NO pathway in ECs.^424–426^ Clinical studies showed that reduced circulating Apelin was significantly associated with an increased risk of hypertension.^427^ And in animal studies, peripheral injection of Apelin can cause extensive vasodilation, as well as a decrease in blood pressure.^428^ Furthermore, Apelin/APJ also interacts with RAAS. Activation of apelin/APJ signaling pathway has an antagonistic effect towards AT1R-mediated responses.^429–431^ Apelin/APJ signaling can also upregulate ACE-2 gene expression, reinforcing the conversion from Ang II to Angiotensin 1–7.^432^ This inhibition of the RAAS system is an important mechanism for Apelin to exert a protective effect on the cardiovascular system.^433^ To be mentioned, when the vascular endothelium is damaged, Apelin can act directly on the APJ of VSMCs, increasing phosphorylation of MLC and causing vasoconstriction and increased blood pressure.^434,435^ It can be seen that the integrity of the vascular endothelium is very important for Apelin to play its hypotensive effect. Except Apelin, recent studies also found another endogenous polypeptide ligand for APJ, Elabela (also named Toddler).^436,437^ Hypertensive patients seemed to have a low level of circulating Elabela, which was strongly associated with hypertension-related vascular damage.^438^ It is currently believed that the function mechanism of Elabela is similar with that of Apelin, both of which exert their function by activating APJ-mediated downstream pathway.^439,440^ However, some studies showed that differences existed regarding the polypeptide structural characteristics and the functioning active groups between Elabela and Apelin.^441^ This suggested that the binding pattern of Elabela and APJ might be different from Apelin, which could be of value in pharmacology development. Both Elabela and Apelin have their latent value in antihypertensive and cardiovascular treatment.

### Na^+^ channels and hypertension

Alterations in circulating Na^+^ concentration contributes a lot to the pathology of hypertension. On the one hand, increased sodium intake in the body can lead to sodium and water retention, resulting in increased pressure to the blood vessel walls. On the other hand, it can trigger vasoconstriction or relaxation via Na^+^ channels.

The Na^+^/Ca^2+^ exchangers (NCX) can link Na^+^ and Ca^2+^ metabolism and act as distal regulators of cytosolic Ca^2+^ levels. There are 2 types of NCXs. By one kind of NCXs, the Ca^2+^ transfer only depends on Na^+^ concentration (NCXs, including NCX1-3). While by the other kind, the Ca^2+^ transfer depends on both Na^+^ and K^+^ concentration (NCKXs, including NCKX 1-6).^442–444^ Both NCXs and NCKXs exist in VSMCs.^442^ The transferring direction of Ca^2+^ (influx or outflux) via NCXs and NCKXs depends mainly on Na^+^, Ca^2+^ (and K^+^) gradients, and the potential across the membrane.^444,445^ In salt-dependent hypertensive rats, the blockage of NCX1 could cause a reduction in cytosolic Ca^2+^ concentration in VSMCs, thus attenuating the Ca^2+^ signaling and resulting in vasodilation and BP decrease.^446^ This indicated that in high salt-intake condition, NCX1 could still exert its function to mediate Ca^2+^ influx. Besides, NCXs in VSMCs appeared to be predominantly present in the plasma membrane adjacent to SR,^447^ which suggested that NCXs in VSMCs might indirectly regulate the Ca^2+^ storage in SR.

Na^+^ pumps (Na, K-ATPase) can transport Na^+^ from intracellular to extracellular and K^+^ in an inverse direction against a concentration gradient by breaking down ATP, maintaining the osmotic pressure across cellular membrane. Theoretically, inhibition of the Na^+^ pumps activity will attenuate Na^+^ transporting in renal proximal tubular epithelial cells, leading to an increase in water sodium excretion and downregulating BP.^448^ Nevertheless, studies showed that inhibition of Na^+^ pumps was a main cause of increased peripheral vascular resistance in essential hypertension.^449^ The mechanisms by which Na^+^ pumps activity enhancement causes vasodilation may be related to both nitric oxide- and prostanoid-independent arterial relaxation,^450,451^ and Ca^2+^ elevation via NCXs.^452–454^ In addition, Na^+^ pumps can regulate inter-cell communications in vessels via cSrc-dependent Cx43 tyrosine phosphorylation, and synchronize the constriction and relaxation among VSMCs.^455^

Epithelial Na^+^ channel (ENaC), a member of DEG/ENaC family, is primarily expressed on the apical membrane of the principal cells in the aldosterone sensitive distal nephron (ASDN), where it functions as the final and rate-limiting step of renal Na^+^ reabsorption. Excessive ENaC activation will lead to sodium and water retention, contributing as a key mechanism to the pathology of hypertension.^456^ In vascular ECs, EnNaC activation by ALD or elevated Na^+^ concentration can lead to endothelial stiffness, reduced NO production, increased vascular tension and vascular remodeling.^457–460^ In CNS, over-activation of ENaC and Na^+^ transport may lead to elevated sympathetic activity and BP increase.^461,462^ In dendritic cells, increased Na^+^ influx mediated by ENaC can promote the release of inflammatory cytokines including IL-17, and therefore upregulate BP.^463,464^ In addition, a recent study demonstrated that the activation of the NLRP3 inflammasome induced by Na^+^ is dependent on ENaC and IsoLG(isolevuglandins), and this was an important mechanism in the pathogenesis of sensitive hypertension.^463^

### Sodium-glucose co-transporter and vascular function

Sodium-glucose co-transporter (SGLT) family refers to carrier proteins that reabsorb filtered glucose in kidneys. It is estimated that SGLT-2 is responsible for at least 80–90% filtered glucose reabsorption.^465^ Meta-analyses showed that SGLT-2 inhibition lowered SBP by 2–5 mmHg and DBP by 0.5–2 mmHg, which might have a coordinated effect with other first-line antihypertensive drugs.^466–468^ The mechanisms by which SGLT-2 inhibition lowers BP include volume depletion,^469–471^ negative sodium balance,^472,473^ weight loss,^474^ renal protection, etc.^475,476^ Moreover, SGLT-2 inhibition can also regulate BP by affecting vascular fuctions. Both clinical RCTS and basic research have shown that SGLT-2 inhibition improves endothelial function as well as the vascular stiffness (measured by PWV, augmentation index, and degree of vascular fibrosis) in DM.^477–480^ Potential vascular protective effects may be attributed to its alleviation of glucotoxicity by removing excessive glucose from the body, as SGLT-2 inhibitors can reduce AGE/RAGE signaling, oxidative stress levels, and the expression of inflammatory factors.^480–482^ In addition, it has been suggested in some studies that SGLT2 inhibitors may suppress sympathetic activity, owing to improved glycemic control and insulin resistance as well as the decreased leptin levels.^483^ However, this mechanism remains controversial since several studies suggested that SGLT-2 inhibitors did not affect HRV or plasma adrenergic markers.^479,484^

### Central nervous system

Initially, it was thought that the central nervous system (CNS) played a limited role in regulating BP, mainly through the baroreflexes and chemoreflexes mechanisms.^485,486^ In fact, the CNS also orchestrates the sympathetic outflow and integrates peripheral inputs.^487^ There are numerous brain nuclei involved in regulating sympathetic tone and BP, and the connections and functional interactions among these nuclei are highly complex. The brainstem and hypothalamus regions form circuits that mediate steady-state and reflex-induced changes in sympathetic activity and BP regulation.^488^ Studies have revealed various mechanisms involving the interactions and functional connections among different cell types in the forebrain and brainstem. Recent findings suggest that Ang-II in the brain may have an impact on bone marrow-derived hematopoietic stem and progenitor cells, potentially exacerbating hypertensive vascular pathological changes.^489–491^ Obesity and high-fat diets are thought to promote a chronic state of low-grade inflammation in the CNS. This is characterized by increased activation of microglia and astrocytes, as well as increased expression of genes encoding pro-inflammatory cytokines (TNF-α, IL-6, and IL-1β).^492^ Pro-inflammatory cytokines elevation in CNS also contributes to SNA activity upregulation and the occurrence of hypertension.^493^

## Interventions

Clinical studies have validated that targeting some vascular function-related pathways (e.g., calcium pathway, RAAS pathway, etc.) for blood pressure control is safe and effective. However, more evidence is needed to determine whether other therapeutic targets (e.g., inflammation, oxidative stress) can be utilized, too. We list below some interventions that target these pathways, both those used in clinical work already and those with potential values (Table 1).

**Table 1:** Drugs/Therapies targeting the related signaling pathways

Targeting signaling pathway	Drug/therapy	Applications	Citations
NO-(NOsGC)-cGMP signaling	Sodium nitroprusside	Clinical use/human	^494^
	Nitroglycerin	Clinical use/human	^494^
	Statins	Mice/rat cells in vitro	^495,496^
	ARBs	Mice/rat cells in vitro	^497^
	Tetrahydrobiopterin (BH4)	Mice/rats/human	^498,658^
	NosGC activators and stimulators	Clinical use/human	^494^
	Resveratrol	Cells in vitro/animals	^499,500^
	ITI-214	Animals/human	^504^
Calcium signaling pathway	Dihydropyridinic agents	Clinical use/human	^659^
	Phenilalchilaminic agents	Clinical use/human	^514^
	Benzothiazepinic agents	Clinical use/human	^660^
Renin-angiotensin-aldosterone system	ACEi	Clinical use/human	^512,513^
	Compound 21	Animals	^527^
	CYT006-AngGb vaccine	Human	^528^
	ATRQ*β*-001 vaccine	Ang II-induced hypertensive mice / SHR	^529^
	Aliskiren	Clinical use/human	^530,531^
	ACT-178882	Human	^534^
	Spironolactone	Clinical use/human	^535^
	Eplerenone	Clinical use/human	^536^
	Canrenone	Clinical use/human	^537,538^
	Finerenone	Human	^539,540^
	BR-4628	In vitro	^541^
	PF-3882845	In the Dahl salt sensitive preclinical model	^542^
	SM-368229	In aldosterone/salt-treated rats	^543^
	FAD 286A	In vitro	^545^
	LCI699	Human	^545^
	Dicer-dependent miRNAs	In vitro	^545^
ACE2–Ang (1–7)–MAS1 axis	ARBs	Clinical use/human	^518^
	ACEi	Clinical use/human	^517^
	Human recombinant ACE2	Human	^524^
	AVE 0991	Rats with 2K1C renovascular hypertension	^525^
	IRAP inhibitors	In vitro	^526^
Angiotensin-receptor–neprilysin	LCZ696 (Sacubatril/Valsartan)	Clinical use/human	^519–522^
MMPs	Statins	Human	^551^
	ARBs	Human	^549^
	ACEi	Human/cells in vitro	^548^
	CCBs	Human/rat	^550^
	Marimastat	Mice	^661^
	Ilomastat	In vitro	^662^
	Batimastat	Animals/human	^556^
	Tanomastat	Rats	^558^
AGEs	Statins	Human/mice	^569^
	ARBs	Human	^569,663^
	ACEi	Human	^563,569^
	Aminoguanidine	Rats	^561^
	Vitamins	Mice	^562^
	Metformin	Human	^564^
	Alagebrium	Human	^567^
	ALT-711	Animals/mice	^664,665^
	Recombinant sRAGE	Rats	^570^
Calcification	Metformin	Rats	^579^
	Exogenous pyrophosphate	Mice	^571–573^
	SNF472	Human/rats	^578,579^
	Valporic acid	In vitro	^582^
	Rapamycin	In the DBA/2 diabetic mouse model	^583^
	XBP1u	Human/mice	^584^
	Zinc	Human	^571–573^
Redox signaling pathway	ARBs	Human/mice/rats/cells in vitro	^594,595,602^
	Resveratrol	Human	^666^
	Dihydropyridinic agents	Cells in vitro	^596,597^
	ACEi	Hypertension models	^594,595^
	Vitamins E	Human	^587,588^
	Vitamins C	Human/mice	^589–591^
	Genistein	Cells in vitro	^598^
	NAC	Human	^667^
	Allopurinol	Human/rats	^600^
Immunity pathway	Statins	Human/cells in vitro	^603^
	ARBs	Human	^602^
	ACEi	Human/animals	^602^
	Mycophenolate mofetil	Dahl salt-sensitive rats and SHR	^604–606^
Sympathetic dysregulation	Selective α1-adrenoceptor antagonists	Clinical use/human	^668^
	Nonselective α-adrenergic antagonists	Clinical use/human	^618^
	α2-adrenergic agonists	Clinical use/human	^669^
	Imidazoline-1 receptor agonists	Clinical use/human	^619,621^
	β-adrenergic receptor antagonists	Clinical use/human	^622^
	Renal denervation	Human	^625^
	Baroreflex activation therapy	Human	^626^

*MMPs* matrix metalloproteinases, *AGEs* Advanced Glycation Endproducts, *ACEi* angiotensin converting enzyme inhibitors, *ARB* angiotensin receptor blocker, *CCBs* calcium channel blockers, *NAC* N-acetylcysteine, *XBP1* X-box-binding protein 1

### NO-(NOsGC)-cGMP signaling

Nitrates, such as sodium nitroprusside and nitroglycerin, are widely used in clinical settings to promote NO production and vasodilation via NO-(NOsGC)-cGMP signaling. However, nitrates commonly have short-term efficacy, and the poor tolerance limits their further use.^494^ Some drugs, reported in animal models, such as statins and Ang II receptor blockers (ARBs) may activate eNOS or increase eNOS expression to improve endothelial dysfunction.^495–497^ In addition to these clinically used drugs, other antihypertensive drugs targeting NO-(NOsGC)-cGMP signaling pathway are still under exploration. As tetrahydrobiopterin (BH4) is a co-factor in eNOS activities, its supplementation may directly activate and regulate the eNOS signaling, which has attracted extensive attention.^498^ NosGC activators and stimulators are currently in clinical use or undergoing clinical development, and may potentially be tested in hypertensive patients in the near future.^494^ Some eNOS transcription enhancers, such as trans-resveratrol, are reported for their therapeutic potential as well.^499,500^ Phosphodiesterase (PDE) is an important substance that degrades intracellular cGMP. PDEs are classified into 11 primary isoenzyme subtypes, which are distinguished by their substrate affinity, selectivity, and regulation mechanisms.^501^ A specific and differential function in contractile VSMCs makes PDE1 inhibition an attractive novel option.^502,503^ Of note, ITI-214, a specific PDE1 inhibitor, has been well-tolerated by humans and a phase 2 clinical trial for heart failure has been completed.^504^

In addition, it is reported that lifestyle interventions should be preliminary approaches for the improvement of NO bioavailability, including a healthy diet, exercise, weight reduction, and smoking cessation. In dietary interventions, supplementation of nitrate and nitrite can exert antihypertensive effects.^505^ Another example is that cocoa can increase NO production, with a beneficial effect on hypertension.^506^ Physical activity can improve downstream NO bioavailability, both in healthy subjects and in those who are at higher cardiovascular risk, to improve endothelial function.^507,508^ Weight reduction^509^ and smoking cessation^510^ are also showed to be helpful for improvement of NO bioavailability.

### Calcium channel signaling

Calcium channel blockers (CCBs) are effective drugs to inhibit calcium influx. Based on their selectivity for L-type voltage-dependent transmembrane calcium channels in either the cardiac, vascular, or both tissues, CCBs can be divided into 3 groups: dihydropyridinic agents, phenilalchilaminic agents, and benzothiazepinic agents. Dihydropyridinic agents can block the voltage-dependent L-type calcium channels, which gives it vascular selectivity to preferentially block the calcium channels in VSMCs. Dihydropyridinic CCBs are now a first-line therapeutic option to treat hypertension.^511–513^ Whereas verapamil and diltiazem, the representative drugs of the latter two types, have cardiac selectivity.^514^ CCBs are known to be vasodilators of small resistance arteries. When administered acutely, they can reduce total peripheral resistance and mean BP, while also increasing cardiac output. However, after chronic administration, cardiac output returns to pretreatment levels, while mean arterial pressure and systemic vascular resistance remain low.

### RAAS

#### Targeting at angiotensin-related receptors

Four groups of drugs are clinically established to inhibit the RAAS: ACE inhibitors (ACEI), ARBs (which block the AT1 receptor), renin inhibitors and mineralocorticoid receptor blockers. Up to now, there are more than 10 types of ACEI available around the world. The main mechanism of ACEI is to limit the formation of Ang II as well as to reduce the catabolism of other vasodilator oligopeptides (such as bradykinin) by ACE. ACEI mediates the activity of the ACE2–Ang (1–7)–MAS1 axis as well.^515,516^ Long-time application in clinical settings have confirmed the safety and tolerability profiles for these drugs. In addition, there exist about nine ARBs for clinical use. ARBs can lead to an increase in the levels of Ang II, which can serve as a substrate of ACE2. This can result in clinical benefits due to the stimulation of the ACE2/Ang (1–7)/Mas1 axis.^515,517^ LCZ696 (Sacubatril/Valsartan) combines a neprilysin inhibitor moiety with a valsartan moiety, and is the first agent of the angiotensin-receptor–neprilysin (ARN) inhibitor. It can decrease BP in animals models and healthy human subjects with low incidence of adverse effects, which has been confirmed by several trials.^518–521^ Notably, LCZ696 is the first drug to be approved for the treatment of heart failure (HF). Clinical trials have shown that LCZ696 is superior to enalapril (an ACEi agonists) in reducing hospitalizations in worsening HF patients and all-cause mortality in patients with left ventricular ejection fraction (LVEF) ≤40%.^522^

As for the ACE2/Ang (1–7)/Mas1 axis, Human recombinant ACE2,^523^ AVE 0991,^524^ and IRAP inhibitors^525^ are considered as promising drugs in hypertension treatment. Compound 21 is an AT2 agonist which can alleviate hypertension and target-organ damage.^526^ To be mentioned, there are currently vaccines under development that target the RAAS. For example, the CYT006-AngGb vaccine, which targets Ang II, has shown promising results in a phase II clinical trial by reducing BP in patients with mild to moderate hypertension without causing serious adverse events.^527^ The ATRQ*β*-001 vaccine, which targets the AT1R, has shown success in lowering BP in Ang II-induced hypertensive mice and SHR.^528^ However, more further studies on antihypertensive vaccines are warranted. However, vaccines may cause pain and require repeated injections,^527^ while oral medication is mature and painless.

#### Targeting at renin and MR

In theory, blocking the rate-limiting enzyme, renin, can be more effective in preventing Ang II production compared to other approaches that target different components of the RAAS. Currently, the only available direct renin inhibitor for the treatment of hypertension is Aliskiren, which is an orally active non-peptide drug with high selectivity.^529–531^ However, it did not achieve satisfying effects in clinical trail.^242^ And another drug, ACT‑077825, lacks evidence to confirm its effect. ACT 178882, the next generation of ACT‑077825, is still under exploration.^532,533^

The mineralocorticoid receptor antagonist mainly refers to spironolactone, eplerenone, and canrenone. As a potassium-sparing diuretic, the efficacy of spironolactone to treat hypertension has been confirmed by RCTs.^534^ Eplerenone has a similar antihypertensive efficacy as spironolactone, but with fewer adverse effects. However, the evidence is yet relatively insufficient.^535^ Canrenone is available in some countries, but there is no large RCT demonstrating its beneficial effect.^536,537^ Some other drugs, such as finerenone, a nonsteroidal mineralocorticoid receptor antagonist, has presented a promising effect in hypertension treatment.^538,539^ BR-4628,^540^ PF-3882845^541^, and SM-368229^542^ are nonsteroidal drugs still under exploration at present. Further studies are needed to confirm their safety and efficacy.^543^ As for drugs targeting at aldosterone synthase, FAD 286A, LCI699 and its second-generation drug are still under development.^544^ Besides, the expression of CYP11B1 and CYP11B2, which are responsible for the production of aldosterone and cortisol in adrenocortical cells, can be regulated post-transciptionally by dicer-dependent microRNAs (miRNAs), affecting the secretion of these hormones.^545^

### Anti-vascular remodeling therapy

#### MMPs

Antihypertensive drugs, including ACEI, ARBs, and CCBs, can regulate the MMP activity and concentration.^546–549^ Administration combined with atorvastatin for 2 months induced a larger reduction in MMP-9 compared with administration alone in hypercholesterolemic subjects.^550^ To be noted, there are studies confirming an improvement in MMP reduction after weight loss and appropriate exercise.^551–553^

MMPs inhibitors can be classified into two categories, hydroxamate-based inhibitors and non-hydroxamate MMP inhibitors. Collagen-based peptidomimetic hydroxamates include marimastat, ilomastat, and batimastat. Batimastat is the first MMP inhibitor studied in clinical trials. It is a low-molecular-mass hydroxamate derivative with low water solubility.^554^ It exerts its inhibitory effect by directly binding to Zn^2+^ ions in the active site of several MMPs, including MMP1, MMP2, MMP7, and MMP9.^555^ Non-hydroxamate MMP inhibitors, including rebimastat, tanomastat, etc. Rebimastat is a broad-spectrum MMP inhibitor which contains a thiol zinc-binding group.^556^ Tanomastat contains a thioether zinc-binding group and a biphenyl deep-pocket-binding segment, and it has been confirmed in trials to be well-tolerated.^557^ However, no specific MMPs inhibitors have been used to treat vascular remodeling or hypertension. In treatment of cancer, despite the promising preclinical data supporting the use of MMP inhibitors as anticancer drugs, it failed to achieve the desired results in clinical settings.^558^ Similarly, more evidence is needed for the utilization of MMPs inhibitors in the treatment of hypertension.

#### AGEs

Levels of AGEs can be reduced by comsuming a diet low in AGEs, which involves reducing the intake of glucose, red meat, butter, cream, and other sweetened fatty foods but increasing the proportion of grains, vegetables, fruits, and milk in the diet.^559^ From the perspective of AGE formation, there are drugs such as aminoguanidine,^560^ vitamins,^561^ ACEI,^562^ metformin,^563^ acidic ingredients,^128^ and pomegranate that can suppress its production.^564^ Animal studies and clinical trials proved that, alagebrium and ALT-711 could degrade AGE.^564–567^ RAGE expression can be suppressed by numerous agents, such as statins, ACEI and ARBs.^568^ Besides, statins, ACEI, ARBs, antidiabetic drugs, and systemic administration of recombinant sRAGE have been reported to elevate the levels of both sRAGE and esRAGE.^569^

#### Calcification

An effective measure to prevent calcification in murine models is the administration of exogenous pyrophosphate.^570–572^ Moreover, it has been shown that inhibition of TNAP (tissue-nonspecific alkaline phosphatase)^573–575^ and exogenous administration of ENPP1^576^ can also prevent vascular calcification. Nevertheless, the effects of these treatments still need to be further discussed in humans. SNF472 is an intravenous formulation of myo-inositol hexaphosphate. It can inhibit the formation and growth of hydroxyapatite crystals through a novel pathway, which is the final common step in the pathophysiology of vascular calcification, selectively and directly.^577,578^ Ferroptosis has emerged as a potential therapeutic target for anti-calcification intervention. Metformin can exert anti-ferroptotic effects and attenuate hyperlipidemia-associated vascular calcification.^579^ Moreover, recent evidence suggests that autophagy may have a direct protect effect against vascular calcification.^580^

There are also some other potential drugs or treatment targets. For example, Valporic acid, an inducer of autophagy, has been demonstrated to inhibit VSMC calcification in vitro.^581^ Rapamycin has been shown to alleviate vascular calcification in DBA/2 mice with diabetes.^582^ XBP1u (unspliced X-box binding protein 1), an endogenous inhibitor, can promote β-catenin ubiquitination degradation and attenuate vascular calcification.^583^ Suppressing activation of NF-κB, zinc can potentially protect against phosphate-induced arterial calcification. Furthermore, there is evidence to suggest that higher intake of dietary zinc is related to a reduced risk of severe abdominal aortic calcification.^584^

### Anti-oxidative stress therapy

Evidence is mounting that antioxidants may paly a crucial role in the management of hypertension. They have also been shown to improve endothelial function, attenuate vascular remodeling, and lower arterial stiffness in some models.^585,586^ Antioxidants compounds such as vitamins E (or α-tocopherol) and vitamins C (or ascorbic acid), polyphenols and some clinical hypertensive drugs may have antioxidative pleiotropic effects. Vitamins E is a promising drug with its potenial as an antioxidant. Several clinical trials have demonstrated its important role in prevention of hypertension.^587,588^ Vitamins C is a potent water-soluble antioxidant which may improve vasodilation response in hypertension by increasing eNOS activity and reducing ROS levels.^589–591^

Polyphenols have been shown to have antihypertensive effects and to improve endothelial function, which may be due to its ability to inhibit ROS generating enzymes and to enhance GSH.^592,593^ RAAS inhibitors such as ACEI and ARBs can effectively lower NADPH oxidase levels. In addition, treatment with ACEI and ARBs has been associated with an increase in superoxide dismutase activity.^594,595^ Antioxidant effect of dihydropyridine CCBs can contribute to the prevention and medication of endothelial dysfunction as well. Nifedipine and nicardipine have been demonstrated to prevent ROS-induced endothelial dysfunction directly and improve endogenous antioxidants in cultured cell lines.^596^ Benidipine has a protective effect on human endothelial cells via reducing oxidative damage induced by ox-LDL which triggers ROS generation.^597^ There are also some drugs that have shown antioxidant and antihypertensive effects in studies, such as Genistein,^598^ N-acetylcysteine(NAC),^599^ and Allopurinol.^600,601^ Nevertheless, their functions need to be further elucidated.

### Anti-inflammation

RAAS-suppressing drugs have been shown to possess potent anti-inflammatory effects. It is noteworthy that, such anti-inflammatory effects may be unrelated to their BP-lowering effects and instead may be due to their ability to directly counteract the pro-inflammatory effects induced by Ang II.^602^ Statins may have anti-inflammatory effects and can lead to a small decrease in systolic BP in patients with hypercholesterolemea, by reducing pro-inflammatory cytokines levels.^603^ Some immunosuppressant drugs have potential as a treatment for hypertension, too. Mycophenolate mofetil has been found to reduce BP in SHR,^604^ and in Dahl salt-sensitive rats.^605,606^ Lifestyle also affects BP by alleviating inflammation. Mediterranean diet is closely related to anti-inflammatory effects and endothelial function.^607^ A regular and moderate aerobic physical exercise can also present anti-inflammatory effects.^608^ However, whether anti-inflammation can be used to treat hypertension clinically it is still doubtful. Nonsteroidal anti-inflammatory drugs can lead to an increase in BP by promoting sodium retention.^609,610^ Furthermore, Canakinumab, the anti-IL-1β monoclonal antibody, reduced inflammatory factors including IL-1β, IL-6, hs-CRP in patients with atherosclerotic disease, but it did not reduce the incidence of hypertension nor down-regulated BP at 3, 6, or 12 months.^364,611–614^ However, study suggested that plasma IL-1β concentration elevated in hypertensive patients, no significant change was observed in its receptors levels. And it indicated that, without IL-1β-related indicators of cellular immune activation, the reduction in plasma IL-1β level alone might be insufficient to reflect the levels of other inmmune activatioin.^615^ In addition, inflammation in hypertensive patients is considered to be low-level and chronic inflammatory stimuli. The longest follow-up duration in the CANTOS study was 12 months. It is rather unclear when vascular remodeling occurs in patients with hypertension. The 1-year period seems to be relatively short, so the follow-up time of this study may be responsible for the contradiction. These may be the reasons why anti-IL-1β failed to reduce BP. In all, specific anti-inflammatory drugs did not achieve significant antihypertensive effects yet in clinical studies. Whether hypertension can be treated with anti-inflammatory drugs remains controversial.^616^

### Sympathetic nervous system

Clinically, it is one of the important means to control BP by restricting the effect of released NE, with drugs competitively binding to adrenergic receptors. Selective α1-adrenoceptor antagonists mainly include prazosin, terazosin, and doxazosin. α2-adrenergic agonists, such as clonidine, can stimulate α2-adrenoceptor in the brainstem and subsequently reduce sympathetic outflow from the CNS, leading to a decrease in BP.^617^ Nonselective α-adrenergic antagonists, phentolamine and phenoxybenzamine, were primarily administered parenterally for the management of hypertensive crisis.^618^ The reduction in plasma NE is directly associated with the hypotensive effect. Clonidine can lower BP via cardiac output and total peripheral resistance. Moxonidine and rilmenidine, which are imidazoline-1 receptor agonists, have been shown to be equally effective as other classes of antihypertensive agents, with their mechanism of action involving a reduction in sympathetic outflow from the CNS. In addition, their side effects like sedation, dry mouth, and rebound hypertension, were not commonly observed in studies.^619–621^ β-adrenergic receptor antagonists, like propranolol, can lower BP by decreasing myocardial contractility, heart rate, and cardiac output. Moreover, β-adrenergic receptor antagonists can improve the prognosis of chronic HF, coronary heart disease and other cardiovascular diseases. Therefore, β-adrenergic receptor antagonists are often used as first-line drugs for hypertension complicated with chronic HF and coronary heart disease.^622^

Intervening sympathetic activity by invasive means can also achieve the purpose of controlling BP. Surgical interventions such as thoracic sympathectomy were already abandoned due to their high mortality rate and relatively high incidence of complications.^623^ Recent treatments for hypertension have focused on device-based approaches, such as renal denervation and baroreflex activation therapy, to modify the SNS. Although renal denervation seemed fail to cure resistant hypertension, based on the sobering results from SYMPLICITY-HTN 3,^624^ it remains a promising therapeutic method with huge potential.^625^ Baroreflex activation therapy is also considered as an alternative choice for resistant hypertension.^626^

Lifestyle interventions can reduce SNA and BP in hypertensive patients as well. Neurogenic components contribute much to obesity-related hypertension.^627^ Exercise training and/or low caloric intake can lead to weight loss and down-regulate SNA.^628,629^ The effects of exercise involve up-regulating central antioxidants concentrations, reducing pro-oxidant levels, and increasing central nitric oxide synthase activity.^630^ Chronic psychosocial stress is also associated with highly activated SNS and abnormal BP.^631^ Stress reduction measures are supposed to be effective in reducing SNA and lowering BP in hypertensives. Besides, device-guided, home-based slow and deep breathing training has been shown to effectively reduce BP in patients with hypertension as well.^632–634^

## Biomarkers

Biomarkers can be used to predict disease development, identify disease, define disease severity, and assess prognosis and treatment effect.^635^ The application of biomarkers in clinical settings contributes greatly to disease monitoring and prognosis prediction. For example, tradional markers like CRP can be used to predict the incidence of hypertension,^636^ sortlin is able to predict the onset of vascular dysfunction and hypertension,^637^ APCs and Klotho can be used to identify salt-sensitive hypertension,^463,638^ platelets and circulating CD34-positive cells can be important indicators of vicious cyclical activity between hypertension and endothelial dysfunction,^639^ and high-sensitivity cardiac troponin T or NT-proBNP can better guide patients in BP control after being incorporated into risk assessment algorithms.^640^ As for urinary microalbumin, it is not only applied to BP monitoring, but also to the hypertension-related risk of cardiovascular diseases and mortality assessment.^641^

Except predicting the initiation and progression of diseases, it is also promising to determine potential treatment targets in the biomarkers-related signaling pathways. Sortlin is a member of the vacuolar protein sorting 10 (VPS10P) family of receptors.^642^ It was recently reported to induce endothelial dysfunction of mesenteric arteries via activating NOX2 (NADPH oxidase 2) isoform, which could be prevented by ASMase (acid sphingomyelinase) or sphingosine kinase 1 knockdow.^637^ Addtionally, patients with hypertension, especially those with uncontrolled BP, exhibited an increase in plasma ASMase activity and plasma sortilin concentration.^637^ Similarly, some other newly discovered circulating proteins (such as Sphingosine-1-Phosphate,^643^ Klotho^644^) and some small molecules (such as non-coding RNAs,^645,646^ microvesicles^647^) can function as predicting biomarkers, as well as potential treatment targets.

## Conclusion and perspectives

An updated Mosaic Theory has been proposed to explain the pathogenesis of hypertension. According to this theory, hypertension is believed to be a response to various combinations of traits and stressors.^648^ The new Mosaic Theory emphasizes other factors such as oxidative stress, sympathetic activation, inflammation, genetics, microbiome, renal mechanisms, and salt intake, in addition to vascular fuction.^648^ Although the interaction of these factors results in a complex pathogenesis of hypertension, resembling a network, vascular function is undoubtedly the direct cause of BP elevation. BP control in clinical practice is mainly based on targeting vascular function, such as RAAS inhibitors, CCBs, nitrates, etc. On the contrary, the therapeutic potentials of other factors, except renal function, have not been well elucidated in clinical use or in large clinical studies.

**Fig. 9:**
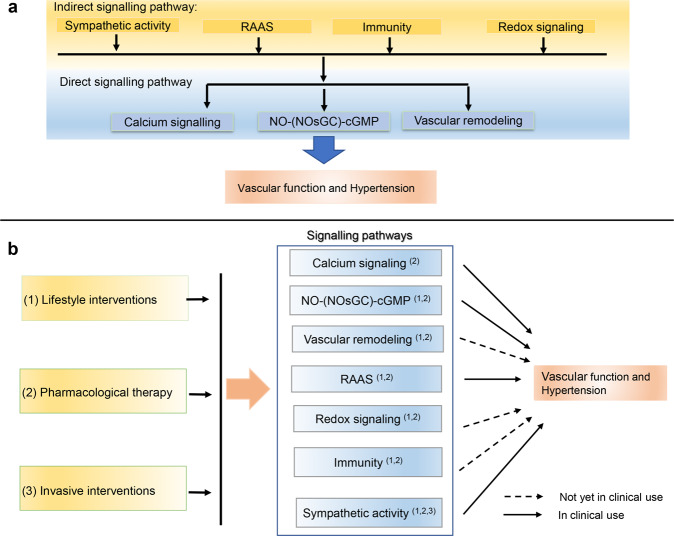
Molecular pathways and therapeutic strategies in vascular function and hypertension. **a** Molecular pathways that act on blood vessels. **b** Therapeutic strategies targeting blood vessels. ^(1)^, ^(2)^, and ^(3)^ in the signaling pathways section represent the specific intervention involving each pathway according to current evidence

In vascular function, the interaction network formed by molecular pathways is also complex. We divide the molecular pathways that act on blood vessels into two categories (Fig. 9a) One indirectly affects vascular sympathetic activity, such as RAAS, immunity, and redox signaling. The other directly affects vascular functions, such as calcium signaling, NO-(NOsGC)-cGMP, vascular remodeling, etc. The former type of interaction has a complex network and mainly induces vasoconstriction by regulating the direct pathway, for example, sympathetic disorders mainly trigger vasoconstriction by activating calcium channels, RAAS activates calcium channels and triggers vascular remodeling.

Long-term control of BP is currently the main strategy for treating hypertension and reducing target organ damage in hypertension. In therapeutic strategies targeting blood vessels, there are mainly three ways (Fig. 9b): lifestyle interventions, pharmacological therapy, and invasive interventions. Lifestyle interventions, especially diet and exercise, can affect vascular function and BP in almost all pathways. Llifestyle modification is recommended as the first line of antihypertensive treatment.^649,650^ Drug therapy is the basis of BP control in clinical work. RAAS inhibitors, and CCBs are first-line antihypertensive drugs; β-blockers and α-adrenergic antagonists play important roles in the treatment of sympathetic-induced hypertension; Nitrates are commonly used in hypertensive emergencies and sub-emergency. Invasive intervention brings new perspective on the treatment of resistant hypertension. As for the utilization of molecular pathways concerning vascular functions in guiding the treatment of hypertension, the efficacy of calcium signaling, NO-(NOsGC)-cGMP, RAAS, and sympathetic activity have been clearly demonstrated in clinical practice. Nevertheless, drugs targeting vascular remodeling, redox signaling, and immunity are still under studying.

**Fig. 10:**
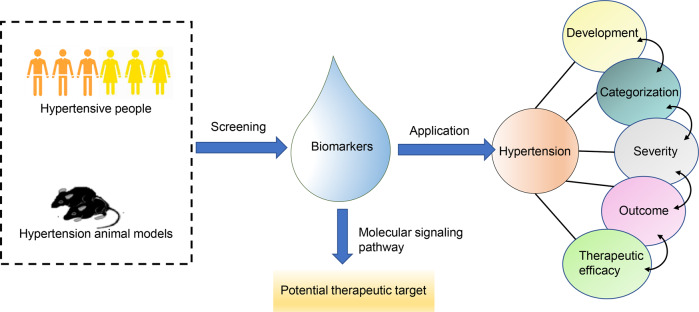
Application of biomarkers in clinical diagnosis and treatment of hypertension

For the next research direction, translating basic study findings on vascular remodeling, redox signaling, and immunity to clinical applications is a priority. Especially vascular remodeling. From the current study, vascular remodeling is difficult to reverse, and it may be closely related to refractory hypertension. Since the state and function of cells (especially secretory function) directly affect the ECM, the elucidation of cell identity and the rewriting of cell fate may be crucial to make a breakthrough in vascular remodeling. Therefore, completing vascular cell maps in different pathological conditions should be one important direction in further studies. On the other hand, although current antihypertensive drugs are effective, many people with hypertension still struggle to achieve target BP levels,^651^ and the optimal BP goal also remains controversial.^652–654^ The importance of reducing cardiovascular events should be emphasized in BP control.^655^ Therefore, achieving BP targets while simultaneously preventing cardiovascular events is the main goal in hypertension treatment. As discussed above, searching for suitable biomarkers and forming new diagnosis and treatment protocols is promising to save the problem (Fig. 10). Urinary microalbumin is a successful case of biomarkers applied to monitoring BP control as well as predicting cardiovascular events,^641^ it is now a classic indicator widely accepted by clinical guidelines for hypertension. Utilization of high-throughput sequencing and machine learning are cutting-edge methods to further mine the role of biomarkers.^656,657^

It is worth mentioning that hypertension has been defined as a disease for over a century. However, modern medicine tends to view hypertension as a risk factor for vascular disease rather than a disease in itself. The essence of controlling BP is to reduce cardiovascular events, similar to controlling blood lipids and glucose. We need to be aware that the molecular mechanism of hypertension is essentially a molecular mechanism of vascular dysfunction and/or vascular volume. Understanding this is particularly important for in-depth research on the signaling pathways of hypertension.

In conclusion, our review demonstrated the signaling pathways involved in the vascular function and hypertension, and summarized therapeutic methods of hypertension which target at vascular function. In addition, we raised unresolved questions in vascular function and hypertension, and presented our own perspectives. It is expected to enlighten other researchers on the future research directions.
